# Astrocyte Lipid Droplet Dynamics Orchestrate Neurological Disorders and Therapeutic Horizons

**DOI:** 10.1002/smsc.202500152

**Published:** 2025-06-08

**Authors:** Jiani Zhong, Yanyi Peng, Lingyue Zhang, Bo Xiao, Mengqi Zhang

**Affiliations:** ^1^ Department of Neurology Xiangya Hospital Central South University Changsha 410008 China; ^2^ National Clinical Research Center for Geriatric Disorders Xiangya Hospital Central South University Changsha Hunan 410008 China

**Keywords:** astrocytes, lipid droplets, metabolism, neurological disorders, pathogenesis, therapeutics

## Abstract

Astrocytes, the predominant glial cells in the central nervous system (CNS), play a pivotal role in maintaining neuronal homeostasis and function. Accumulating evidence suggests that astrocytic dysfunction is closely associated with the pathogenesis of various neurological disorders, including neurodegenerative diseases, ischemic stroke (IS), epilepsy, and glioma. Lipid droplets (LDs), ubiquitous intracellular lipid storage organelles, exhibit metabolic abnormalities that are commonly observed in these neurological conditions, particularly in astrocytes, where LD metabolic dysregulation may serve as a critical link between glial dysfunction and neuronal damage. However, a systematic understanding of the regulatory mechanisms governing LD metabolism in astrocytes and their relationship to the pathogenesis of neurological diseases remains elusive. This article reviews the biology and pathology of astrocytes and summarizes the characteristics, regulatory factors, and abnormalities of LD metabolism in astrocytes, highlighting its association with neurodegenerative diseases, stroke, epilepsy, and glioma. Finally, we propose future research directions, emphasizing the need for integrative multiomics approaches and innovative regulatory technologies to elucidate the role of astrocytic LD metabolism in neurological disorders. Understanding the dysregulation of LD metabolism in astrocytes may provide novel insights into disease etiology and facilitate the development of glial‐targeted diagnostic and therapeutic strategies.

## Graphical abstract

This review examines astrocyte lipid droplet (LD) metabolism in neurological disorders, including Alzheimer's disease, stroke, epilepsy, and glioma. It analyzes regulatory mechanisms governing LD formation, transport, and degradation in astrocytes, highlighting their potential as biomarkers and therapeutic targets through integrative multiomics

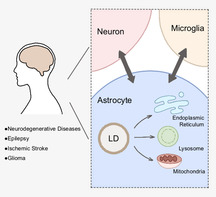

## 1 Introduction

Astrocytes, the most abundant and widely distributed glial cells in the central nervous system (CNS), play a crucial role in maintaining brain homeostasis and supporting neuronal survival and function.^[^ ^1^ ^]^ For instance, astrocytes express glutamate transporters, which participate in clearing excess excitatory neurotransmitter glutamate from the synaptic cleft, thereby preventing its excitotoxicity to neurons. The foot processes of astrocytes envelop the microvasculature and maintain the integrity of the blood–brain barrier (BBB) by secreting tight junction proteins and facilitating the transport of nutrients, thus regulating the supply of substances and energy in the brain^[^ ^1^ ^]^ Furthermore, astrocytes secrete various neurotrophic factors, such as brain‐derived neurotrophic factor (BDNF) and glial cell‐derived neurotrophic factor (GDNF), which directly support neuronal growth, development, and synaptic plasticity.^[^ ^2^ ^]^ However, in various neurological diseases, including neurodegenerative disorders, ischemic stroke (IS), epilepsy, and glioma, astrocytes undergo significant morphological and functional changes, suggesting that astrocytic dysfunction may play a pivotal role in the pathogenesis of these conditions.^[^ ^3^, ^4^, ^5^, ^6^ ^]^

Lipid droplets (LDs), widely present lipid storage organelles in cells, play a vital role in regulating intracellular lipid homeostasis, participating in lipid metabolism, and mediating signal transduction.^[^ ^7^ ^]^ LDs are primarily composed of a neutral lipid core, containing triglycerides (TAGs) and cholesterol esters (CEs), surrounded by a peripheral monolayer phospholipid membrane with various functional proteins embedded on the surface that regulate LD metabolism. The formation, growth, transport, and degradation of LDs are tightly regulated by various intracellular and extracellular factors, and alterations in LD metabolism are often closely associated with the onset and progression of various diseases.^[^ ^8^, ^9^, ^10^ ^]^ It is particularly noteworthy that LDs are widely distributed in various cell types of the nervous system, and their metabolic changes are closely linked to neurological disorders. For instance, a significant increase in LD accumulation has been observed in astrocytes and microglia in the brain tissues of Alzheimer's disease (AD) patients.^[^ ^3^ ^]^ Moreover, LDs amass in the astrocytes surrounding the epileptic foci in the brain tissue of epilepsy patients.^[^ ^4^ ^]^ A substantial accumulation of LDs has also been documented in astrocytes within the infarcted area in animal models of IS.^[^ ^5^ ^]^ These studies suggest that metabolic disorders of LDs, particularly aberrant LD accumulation in astrocytes, may be a crucial link in the onset and progression of neurological diseases.

Given the prevalence and significance of astrocyte dysfunction and LD metabolic disorders in the pathogenesis of neurological disorders, an in‐depth exploration of the underlying mechanisms connecting these two factors is of paramount importance for unraveling the molecular pathological processes of these diseases and developing novel diagnostic and therapeutic strategies. Currently, a growing number of studies are focusing on elucidating the regulatory mechanisms and abnormal alterations of LD metabolism in astrocytes, analyzing the relationship between LD metabolites and astrocyte function as well as neuronal survival, and deciphering the molecular pathways involved in LD metabolic disorders contributing to the pathogenesis of neurological diseases. Concurrently, molecules and pathways related to LD metabolism are gradually being explored as potential biomarkers and therapeutic targets for neurological disorders, providing new avenues for disease diagnosis and drug development.

After briefly introducing the basic biological properties of astrocytes and LDs, this review will focus on investigating the characteristics, regulatory mechanisms, and abnormalities of LD metabolism in astrocytes, analyzing the relationship between LD metabolism and the pathogenesis of neurological diseases, discussing the potential of utilizing LDs as targets for disease diagnosis and treatment, and proposing future research directions in this field. The aim is to provide novel insights and strategies for understanding the etiology, prevention, and treatment of neurological disorders.

## 2 Astrocyte Biology and Pathology

### 2.1 Astrocyte Functions in the Healthy CNS

Astrocytes are highly heterogeneous cells that exhibit diverse morphologies and functions across different CNS regions.^[^ ^11^, ^12^ ^]^ They play crucial roles in brain development, homeostasis, and neuronal support, including neurotrophic support, neurotransmitter uptake and recycling, synapse formation and function, blood flow, and glymphatic regulation and immune modulation.^[^ ^11^ ^]^

When it comes to neurotrophic support, astrocytes can secrete growth factors, like BDNF^[^ ^13^ ^]^ and GDNF,^[^ ^14^ ^]^ to promote neuronal survival and plasticity, assisting neuron formation and function including learning and memory. Besides, astrocytes uptake and recycle neurotransmitters (e.g., glutamate and γ‐aminobutyric acid (GABA) transporters), to regulate synaptic transmission and control neural function.^[^ ^15^ ^]^ Then, astrocytes release gliotransmitters and shaping synaptic connectivity.^[^ ^16^, ^17^, ^18^ ^]^ Astrocytic LD metabolism also regulates synaptogenesis. Lower cholesterol level will lead to defective synaptic plasticity.^[^ ^19^ ^]^ Sterol regulatory element‐binding protein (SREBP)^[^ ^19^ ^]^ and γ‐secretase^[^ ^20^ ^]^ will affect cholesterol level in astrocytes. What is more, cerebral blood flow can be controlled by astrocytes in response to neuronal activity, so as to ensure an adequate supply of glucose and oxygen to meet the metabolic demands of the neurons.^[^ ^21^ ^]^ Astrocytes can astrocytic calcium ion (Ca^2+^)‐dependent signaling pathways, production and release of arachidonic acid metabolites, releasing triphosphate (ATP) and cyclooxygenase (COX)‐1 and so on, to maintain both resting blood flow and activity‐evoked changes.^[^ ^22^ ^]^ Additionally, astrocytes can generate inflammatory factors and release reactive oxygen species (ROS), as well as interacting with microglia and peripheral immune cells to regulate neuroinflammation.^[^ ^23^ ^]^ Astrocytes also exhibit distinct calcium signaling and rhythmic oscillations that contribute to their diverse functions.^[^ ^24^, ^25^ ^]^

### 2.2 Astrocyte Alterations in Neurological Disorders

In neurological disorders, astrocytes undergo complex changes that can be broadly categorized into loss of homeostatic functions and gain of toxic functions.

Loss of homeostatic functions refers to the impairment of astrocytes’ normal supportive roles, such as compromised neurotransmitter uptake, energy metabolism, ion balance, and immune regulation. For example, in AD, astrocytes display reduced glutamate uptake^[^ ^15^ ^]^ and decreased glucose and/or lipid metabolism,^[^ ^26^ ^]^ leading to excitotoxicity and energy deficits. Gain of toxic functions occurs when astrocytes actively contribute to disease progression by releasing neurotoxic factors, such as inflammatory cytokines^[^ ^27^ ^]^ and lipid mediators. In neuroinflammatory conditions, reactive astrocytes secrete proinflammatory molecules^[^ ^28^ ^]^ (e.g., interleukin (IL)‐1β, tumor necrosis factor (TNF)‐α), and chemokines^[^ ^29^, ^30^ ^]^ (e.g., C‐C motif chemokine ligand (CCL) 2, C‐X‐C motif chemokine ligand (CXCL) 10) that exacerbate neuronal damage.

Recent studies have revealed the heterogeneity of astrocyte responses in neurological disorders.^[^ ^31^ ^]^ Reactive astrocytes can be classified into two primary phenotypes based on their transcriptional profiles and functional properties: neurotoxic/proinflammatory (A1) and neuroprotective/anti‐inflammatory (A2) astrocytes.^[^ ^32^, ^33^, ^34^ ^]^ A1 astrocytes^[^ ^35^ ^]^ are characterized by upregulation of complement cascade genes and loss of homeostatic functions, while A2 astrocytes exhibit increased expression of neurotrophic factors and promote neuronal survival. The balance between these astrocyte phenotypes critically shapes neuroinflammatory processes and neurodegeneration in various disease contexts.^[^ ^36^ ^]^

Moreover, astrocytes undergo subtype‐specific transcriptional changes in different CNS regions and disease stages.^[^ ^37^ ^]^ For instance, in AD, astrocytes in the prefrontal cortex and hippocampus display distinct gene expression profiles compared to those in the cerebellum.^[^ ^38^ ^]^ Understanding the regional and temporal heterogeneity of astrocyte responses may provide insights into the selective vulnerability of certain brain areas in neurological disorders.

## 3 LD Biology

### 3.1 LD Structure and Composition

In addition to TAGs and CEs, the neutral lipid core of LDs contains other neutral lipids, such as retinol esters and free cholesterol, depending on the cell type.^[^ ^39^ ^]^ The metabolism of the core components of LDs, particularly cholesterol and free fatty acids (FFAs), currently serves as a promising target for LD‐targeted drug development.^[^ ^5^, ^40^, ^41^ ^]^

LD‐associated proteins (LDAPs) are a multifunctional proteome that regulates various aspects of LD biology, including growth, degradation, trafficking, and signaling.^[^ ^8^ ^]^ LDAPs encompass the perilipin family, alpha/beta hydrolase domain 5 (ABHD5), Berardinelli‐Seip congenital lipodystrophy 2 (BSCL2), and other proteins.^[^ ^42^ ^]^ The perilipin family comprises six subtypes, perilipin (PLIN) 1‐6,^[^ ^43^, ^44^ ^]^ with PLIN1A, PLIN2, and PLIN5 preferentially binding to TAG‐rich LDs, while PLIN1C, PLIN1D, and PLIN4 bind to cholesterol ester‐rich LDs. Under basal conditions, PLIN1 can stabilize LDs, inhibit lipolysis, and reduce TAG hydrolytic activity.^[^ ^45^ ^]^ This regulatory mechanism prevents excessive release of free fatty acids (FAs), which can accumulate in cells and cause lipotoxicity—a pathological state characterized by cellular dysfunction resulting from lipid overload. Lipotoxicity occurs when excess free FAs (FFAs) accumulate in nonadipose tissues, triggering multiple deleterious processes including mitochondrial dysfunction, oxidative stress, inflammatory pathway activation, endoplasmic reticulum (ER) stress, and disruption of insulin signaling. These processes create a branched vicious cycle of self‐driven lipotoxic processes that ultimately lead to cellular damage and death. PLIN2 levels are correlated with LD abundance, and a mutual stabilizing relationship exists between LDs and PLIN2. PLIN2 promotes LD formation and protects TAGs from decomposition through electron transport chain, protein lipoylation pathways, and so on.^[^ ^46^ ^]^ PLIN3 exhibits similar functions to PLIN2 and is commonly expressed. PLIN5 contains a mitochondria (Mito)‐targeting sequence and can be observed in the cytosol, Mito, and at LD‐mitochondrial contact sites.^[^ ^47^ ^]^ Recent studies have revealed that PLIN2, PLIN3, and PLIN5 are expressed at varying intensities in different brain regions, while PLIN1 and PLIN4 are not detected. Notably, PLIN3 is highly expressed in astrocytes. Interestingly, only PLIN2 expression is regulated by age and neurodegeneration and is associated with inflammatory states.^[^ ^48^ ^]^ A recent animal study on locust aging demonstrated that PLIN2‐induced ectopic lipid accumulation promotes muscle aging in social locusts.^[^ ^49^ ^]^

### 3.2 LD Formation and Degradation

The majority of LD components are synthesized in the ER.^[^ ^9^ ^]^ Phospholipids and TAGs are synthesized via the Kennedy pathway,^[^ ^50^ ^]^ while cholesterol in the ER is converted into CEs through sterol O‐acyltransferase (SOAT) 1/2, also named ACAT 1/2, for storage in LDs.^[^ ^41^ ^]^ Diacylglycerol acyltransferase (DGAT), including DGAT1 and DGAT2, mediates the formation of TAGs, which are subsequently deposited into the hydrophobic phase of the ER bilayer. Seipin then regulates the recruitment of neutral lipids into the neutral lipid lens, promoting the growth of nascent LDs. Finally, LDs bud from the ER and grow by acquiring specific proteins.^[^ ^10^ ^]^

**Figure 1:**
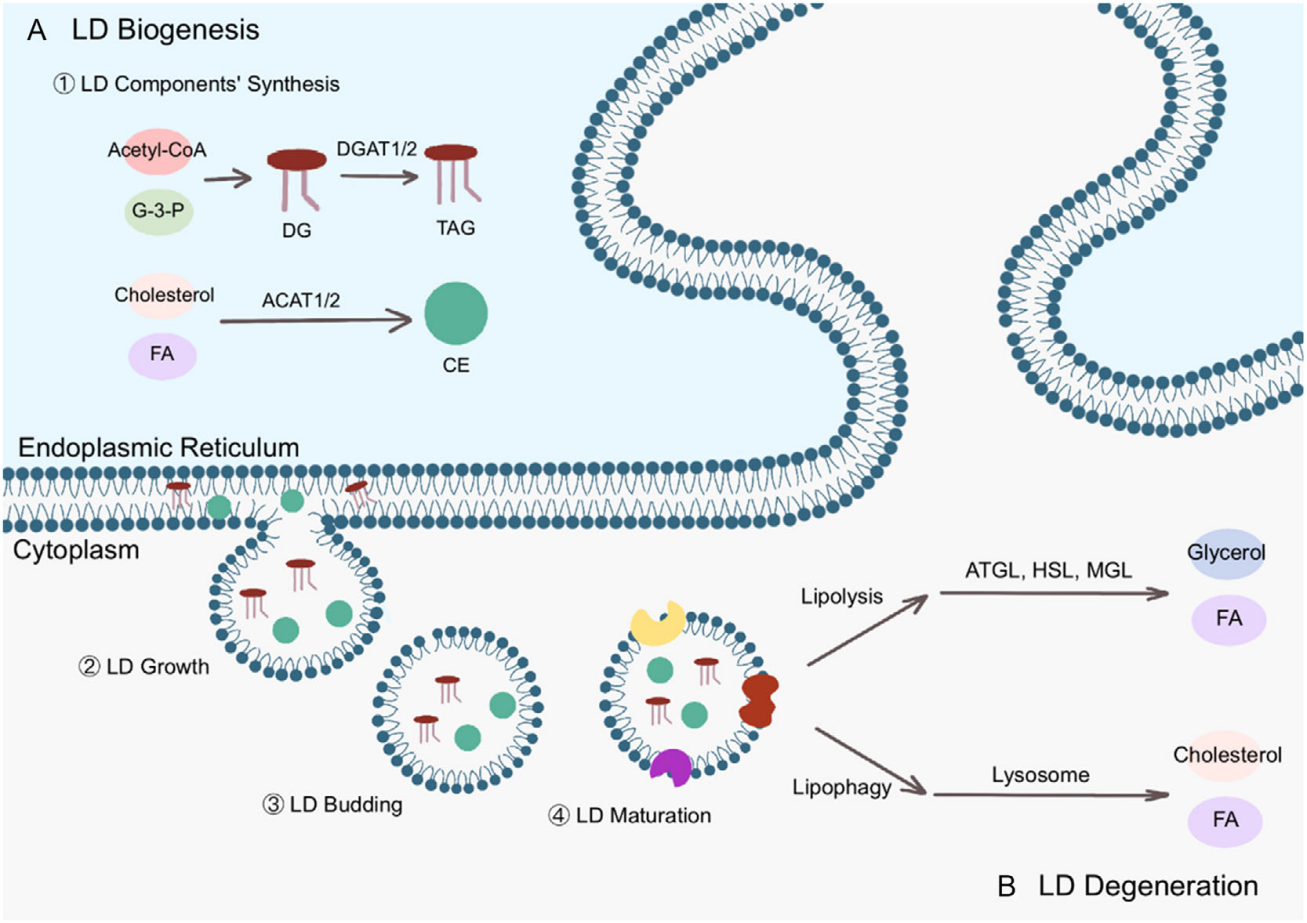
LD biogenesis and degradation. A) LD biogenesis: 1) LD components’ synthesis: exogenous acetyl coenzyme A (acetyl‐CoA) and glycerol‐3‐phosphate (G‐3‐P) are converted into glycerol diesters in the ER, which generate TAGs under the action of diacylglycerol acyltransferase (DGAT) 1/2, while cholesterol and FAs generate sterols (CE) under the action of sterol *O*‐acyltransferase (ACAT) 1/2. Then, TAG and CE are located between the leaves of the ER bilayer and coalescence into a lens‐like structure. 2) LD growth: LD biogenesis factors are recruited to the lens structure, facilitating LD budding from the ER. 3) LD budding: the nascent LD grows and separates from the ER. 4) LD maturation: the LD undergoes maturation while acquiring a coat of proteins. B) LD degradation occurs through two main pathways: lipolysis and lipophagy. Lipolysis sequentially hydrolyzes LDs into glycerol and FAs through the enzymatic actions of adipose TAG lipase (ATGL), hormone‐sensitive lipase (HSL), and monoacylglycerol lipase (MGL). Conversely, lipophagy mediates lysosomal degradation of LDs into cholesterol and FAs.

The degradation of LDs typically involves two processes: lipophagy and lipolysis. Lipophagy refers to the selective engulfment of LDs by autophagosomes for lysosomal degradation.^[^ ^51^ ^]^ The choice between lipophagy and lipolysis appears to be determined by LD size, with smaller LDs being preferentially targeted by the lipophagic machinery.^[^ ^52^ ^]^ Lipolysis and chaperone‐mediated autophagy (CMA) cooperate to reduce LD size, thereby promoting more efficient macrolipophagy. CMA‐dependent degradation of PLIN2 and PLIN3 can facilitate lipase recruitment and LD degradation.^[^ ^9^ ^]^ Recent studies have revealed that the interaction between the lipid transfer protein oxysterol‐binding related protein 8 (ORP8) and microtubule‐associated protein 1 light chain 3 (LC3)/gamma‐aminobutyric acid type A receptor‐associated protein (GABARAP) anchored by phagocytic carriers,^[^ ^53^ ^]^ or the combination of syntaxin 18 (STX18) and autophagy related 14 (ATG14),^[^ ^54^ ^]^ can regulate lipophagy. Lipophagy releases neutral lipids stored in LDs and generates FFAs and cholesterol within cells. In contrast, lipolysis refers to the catabolic metabolism of LDs mediated by lipases.^[^ ^55^ ^]^ Adipose TAG lipase (ATGL), hormone‐sensitive lipase (HSL), and monoacylglycerol lipase (MGL) sequentially mediate the breakdown of TAGs in LDs, ultimately producing FAs and glycerol (**Figure** **1**).

## 4 LDs in Astrocytes

### 4.1 LD Metabolism in Astrocytes

Among the core components of LDs in astrocytes, cholesterol is synthesized de novo, while FAs are derived partly from de novo synthesis and partly from exogenous FFAs that pass through BBB and enter cells via FA‐binding proteins (FABPs). Various stimuli, such as inflammation, oxidative stress, and aging, can induce LD production. Metabolic stress induced by prolonged starvation (>24 h) leads to LD accumulation, characterized by an increase in both the number and size of LDs. Inhibition of DGAT1 and DGAT2 enzymes can prevent hunger‐induced LD accumulation in primary astrocytes.^[^ ^56^ ^]^ During oxygen deprivation in the brain, anaerobic metabolism and glycolysis produce L‐lactic acid, which activates G‐protein coupled receptor 81 (GPR81), downregulates cyclic adenosine monophosphate (cAMP) formation, inhibits lipolysis, and promotes LD accumulation.^[^ ^57^ ^]^

LDs in astrocytes exhibit mobility and can translocate to different organelles and near the plasma membrane for substance exchange or secretion from the cell. An acute increase in intracellular Ca^2+^ levels under ATP stimulation and/or prolonged metabolic stress (e.g., 24‐h nutrient deprivation) can reduce the fluidity of LDs in astrocytes.^[^ ^56^ ^]^ Through their mobility, LDs can associate with mitochondria, undergoing beta‐oxidation and participating in glucose metabolism to produce ATP for energy supply to both astrocytes and neurons. Additionally, LDs can directly release lipids to support axonal growth, synapse formation, and membrane composition.

### 4.2 Regulatory Factors of LD Metabolism in Astrocytes

Astrocytic LD metabolism is regulated by a complex network of factors, including lipid transport proteins, neuronal activity, transcriptional regulators, and hormonal signals. Apolipoprotein E (APOE), the major lipid transport protein in the brain, plays a central role in modulating astrocytic LD metabolism. APOE encapsulates lipoprotein particles and facilitates lipid transport between different cells in the CNS.^[^ ^58^ ^]^ APOE lipoproteins are secreted by brain cells, lipidated by ATP‐binding cassette transporters (ABC) outside the cells, and then internalized through receptor‐mediated endocytosis of lipoproteins. APOE related to LDs has been found to regulate the size and composition of LDs. Unlike the protective effects of APOE2 and APOE3, APOE4 can disrupt lipid metabolism and impair neuron‐ astrocyte coupling.^[^ ^59^ ^]^ Knocking out APOE4 reduces the number of LDs containing more unsaturated TAGs but increases their size, leading to heightened oxidative sensitivity.^[^ ^60^ ^]^ Astrocytes expressing APOE4 show upregulated cholesterol synthesis and metabolism, indicating a correlation with increased cholesterol synthesis and decreased degradation and exocytosis.^[^ ^61^ ^]^ Additionally, choline treatment and inhibition of stearoyl‐CoA desaturase (SCD)‐1 can mitigate the accumulation of LDs in astrocytes.^[^ ^62^ ^]^

Neuronal activity can influence astrocytic LD metabolism through various mechanisms. Glutamate‐induced excitotoxicity in neurons leads to astrocyte LD formation, mediated by APOE‐coated lipoproteins. In response to neuronal excitotoxicity, astrocytes subsequently degrade these LDs and neutralize ROS.^[^ ^40^ ^]^ Reactive astrocytes often appear in close proximity to neuroinflammation and exhibit functional heterogeneity in different neurological diseases.^[^ ^1^ ^]^ Recent studies have demonstrated that reactive astrocytes produce lipoproteins rich in long‐chain saturated FAs. Neuronal uptake of these lipid particles leads to intracellular accumulation of saturated lipids, which disrupts mitochondrial membrane potential and activates caspase‐dependent apoptotic pathways. This specific form of lipid‐induced programmed cell death, termed lipoapoptosis, occurs when excessive saturated lipids overwhelm cellular detoxification mechanisms, ultimately causing neuronal degeneration.^[^ ^63^ ^]^

Under pathological conditions related to neurodegeneration, the LD homeostasis of astrocytes is modulated by adrenergic signaling.^[^ ^64^ ^]^ Exposure to norepinephrine can promote LD accumulation in astrocytes by increasing β‐ and α‐2‐adrenergic receptor (AR) expression and cAMP signaling.^[^ ^56^ ^]^ The activation of β‐ARs in astrocytes stimulates the transcription factor cAMP‐responsive element‐binding protein (CREB). In astrocytes, CREB selectively upregulates the transcription of genes involved in lipid metabolism, promoting LD accumulation.^[^ ^65^ ^]^ Simultaneously, the activation of the β‐AR/cAMP pathway also upregulates L‐lactate production in astrocytes, which in turn triggers further cAMP generation and CREB activation, thereby promoting LD accumulation.^[^ ^57^ ^]^ Furthermore, γ‐secretase has been implicated in the regulation of LD accumulation. Studies have revealed that impaired γ‐secretase activity and the accumulation of amyloid precursor protein (APP) and C‐terminal fragments (CTFs) lead to the dysregulation of liver X receptor (LXR) and cellular lipid metabolism, indicating a functional link between membrane protein hydrolysis and cellular lipid homeostasis.^[^ ^66^ ^]^ More factors can see in **Table** **1**.

**Table 1:** Regulatory factors of LD metabolism in astrocytes. APOE: apolipoprotein E; APP: amyloid precursor protein; AR: adrenergic receptor; cAMP: cyclic adenosine monophosphate; CREB: cAMP‐responsive element‐binding protein; FFA: free fatty acid; MCTs: monocarboxylate transporters; LD: lipid droplet; STAT3: signal transducer and activator of transcription 3.

Metabolism progress	Factors		Possible pathways	Functions	Reference
LD formation	γ‐secretase		APP C99	Lead to decreased uptake of lipoprotein particles and upregulation of endogenous de novo biosynthesis of cholesterol	[66]
	L‐lactate		MCTs and/or ion channels	Lead to excess FFA production and trigger LD accumulation	[57]
	Noradrenaline		β‐AR/cAMP, CREB	Promote astrocyte LD accumulation	[64]
	Reactive astrocytes		–	Produce lipoproteins rich in long‐chain saturated FAs, promote neuroinflammation and neurodegeneration	[145]
LD transportation	APOE	APOE3	–	Regulate LD size and help lipid transportation towards LDs, mitochondria, etc.	[59]
		APOE4	–	Help formation of larger LDs enriched in unsaturated TAGs than APOE3	[59]
LD degradation	Mitochondrial OxPhos inactivity		STAT3	Lead to compromised FA degradation and promote LD accumulation	[68]
	Noradrenaline		α2‐AR	Inhibit lipolysis and promote LD accumulation	[64]

### 4.3 Interactions between Astrocytic LDs and other Organelles

**Figure 2:**
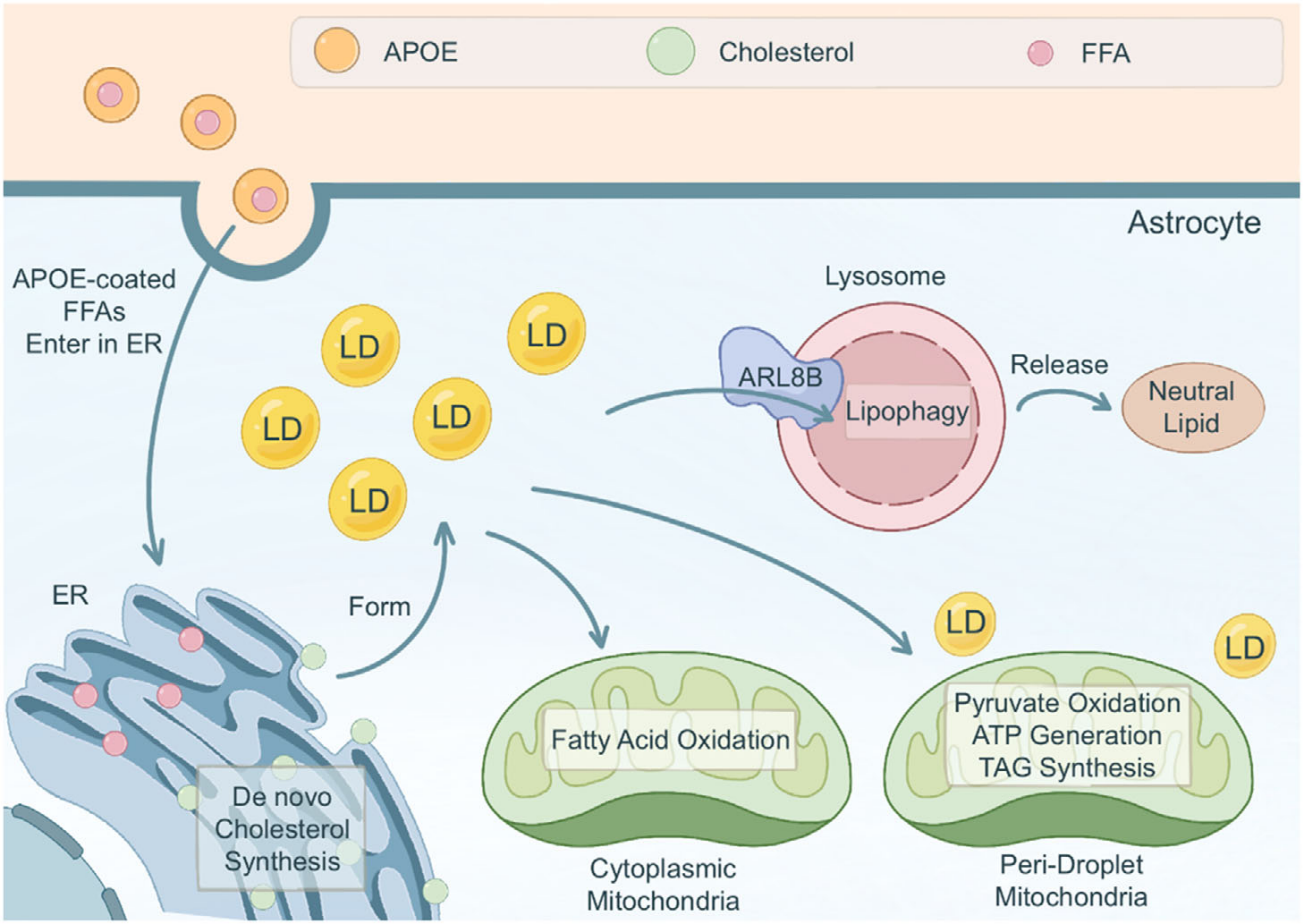
Interactions between LDs and organelles in astrocytes. Apolipoprotein E (APOE)‐coated free FAs (FFAs), typically derived from neurons, enter the astrocyte through endocytosis and are incorporated into the ER bilayer. These FFAs, along with de novo synthesized cholesterol, contribute to the formation of LDs. LDs interact with two distinct populations of mitochondria: cytoplasmic mitochondria and peri‐droplet mitochondria. The interaction between LDs and cytoplasmic mitochondria facilitates FA oxidation, while the association with peri‐droplet mitochondria promotes pyruvate oxidation, adenosine triphosphate (ATP) generation, and triacylglycerol (TAG) synthesis. Additionally, LDs can contact lysosomes through the mediation of ADP‐ribosylation factor‐like GTPase 8B (ARL8B), enabling lipophagy, a process that releases neutral lipids from the degraded LDs.

Astrocytic LDs dynamically interact with various organelles, including the ER, mitochondria, and lysosomes, to coordinate lipid metabolism and cellular functions^[^ ^67^ ^]^ (**Figure** **2**). The ER is the primary site of LD biogenesis, and ER‐LD interactions are direct contact^[^ ^56^ ^]^ or mediated by specific tethering proteins, such as seipin and DGAT2.^[^ ^7^ ^]^ These contacts facilitate the transfer of lipids and proteins between the two organelles and regulate LD growth and maturation. Disruption of ER‐LD contacts can lead to aberrant LD morphology and impaired lipid homeostasis.

Mitochondria play a crucial role in LD catabolism by mediating FA oxidation. LDs can directly associate with mitochondria through protein–protein interactions and membrane contact sites, which are regulated by mitochondrial outer membrane proteins, such as PLIN5 and mitoguardin 2 (MIGA2). These interactions facilitate the transport of FAs from LDs to mitochondria for β‐oxidation and ATP production. Interestingly, a subpopulation of mitochondria, termed peridroplet mitochondria (PDM), has been shown to preferentially associate with LDs and promote LD expansion rather than degradation, suggesting a complex relationship between the two organelles.

Lysosomes are the primary site of lipophagy. As previously mentioned, LDs can be connected to lysosomes through ORP8 and ATG14.^[^ ^53^, ^54^ ^]^ Mitochondria are the site of β‐oxidation. Under starvation conditions, LDs come into contact with mitochondria, promoting the transport of FAs to mitochondria and providing fuel for oxidative phosphorylation. In 2023, Mi et al. demonstrated that the brain heavily relies on astrocyte mitochondrial oxidative phosphorylation (OxPhos) to degrade FAs and maintain lipid homeostasis and that abnormal OxPhos can easily lead to AD.^[^ ^68^ ^]^ Mitochondria also participate in the synthesis of TAGs. The connection between LDs and mitochondria includes protein–protein interactions and direct membrane‐membrane contacts.^[^ ^69^ ^]^ This connection is usually regulated by mitochondrial outer membrane proteins such as Mitoguardin 2 (MIGA2), PLIN5, and DGAT2^[^ ^70^ ^]^ Interestingly, researchers found that PDM are usually more conducive to LD formation rather than decomposition.^[^ ^71^ ^]^ As to peroxisomes, it was observed that LDs and peroxisomes were only rarely in close proximity or direct contact with each other. However, Cheng et al. thought that there may be a network between LDs and peroxisomes, more possibly through an indirect way. VPS13D and RAB, 2 genes that regulate lipid transport and lipolysis, are involved in between LDs and peroxisomes to impact lifespan.^[^ ^72^ ^]^

### 4.4 Crosstalk between Astrocytes and other CNS Cells in LD Metabolism

Astrocytes engage in complex metabolic crosstalk with neurons, microglia, and other glial cells to maintain lipid homeostasis in the CNS.

#### 4.4.1 Astrocyte–Neuron Interactions

Astrocytes and neurons engage in a complex metabolic crosstalk that is essential for brain energy homeostasis and synaptic function.^[^ ^73^ ^]^ One of the key aspects of this metabolic coupling is the astrocyte–neuron lactate shuttle (ANLS).^[^ ^74^ ^]^ Astrocytes predominantly express the glycolytic enzyme 6‐phosphofructo‐2‐kinase/fructose‐2,6‐biphosphatase 3 (PFKFB3), which promotes the production of lactate from glucose.^[^ ^75^ ^]^ The lactate is then transported to neurons through monocarboxylate transporters (MCTs)^[^ ^76^ ^]^ and serves as an energy substrate for OxPhos. Neurons can also utilize lactate to synthesize FFAs, particularly under conditions of mitochondrial dysfunction or oxidative stress.^[^ ^77^ ^]^

In addition to the ANLS, astrocytes and neurons exchange lipids and lipid‐derived signaling molecules that are crucial for synaptic function and plasticity. Astrocytes are the primary source of cholesterol in the brain, which is essential for membrane fluidity, receptor function, and synaptogenesis.^[^ ^19^ ^]^ Astrocyte‐derived cholesterol is transported to neurons via APOE‐containing lipoproteins, which are internalized by neurons through the low‐density lipoprotein receptor (LDLR) and LDLR‐related protein 1 (LRP1). Neurons can also efflux excess cholesterol back to astrocytes through the ATP‐binding cassette transporter A1 (ABCA1), forming a cholesterol shuttle between the two cell types.^[^ ^78^, ^79^ ^]^ Furthermore, the energy supply to the brain mainly comes from lipid metabolism and glucose metabolism, and these two often connect closely. Intermediate products of glucose metabolism, like L‐lactic acid and acetyl‐CoA, can regulate formation, transportation, and catabolism of lipid, controlling lipid‐originated energy generation.^[^ ^80^ ^]^

LDs in astrocytes play a critical role in regulating lipid metabolism and preventing lipotoxicity in neurons. Neurons can accumulate excess FFAs under conditions of high neuronal activity or oxidative stress, which can lead to mitochondrial dysfunction and cell death. To prevent this, neurons transport FFAs back to astrocytes through various receptors, such as APOE, apolipoprotein D (APOD), and FA transport proteins (FATPs).^[^ ^40^, ^81^ ^]^ In astrocytes, these FFAs are either oxidized for energy production or stored in LDs. Astrocytes with abundant LDs express higher levels of genes involved in FFA detoxification and antioxidant defense, suggesting that LDs serve as a protective mechanism against lipotoxicity.^[^ ^82^ ^]^

Interestingly, the metabolic coupling between astrocytes and neurons is bidirectional and can be modulated by neuronal activity. Synaptic transmission triggers Ca^2+^ signaling in astrocytes,^[^ ^83^ ^]^ which in turn stimulates aerobic glycolysis and lactate release. This activity‐dependent regulation of astrocyte metabolism is mediated by various mechanisms, including the activation of glutamate receptors,^[^ ^84^ ^]^ potassium channels, and gap junctions. Currently researchers found that coordination between input from inhibitory neurons and astrocytic GABA_B_ receptor can regulate astrocyte morphogenesis during development, potentially contributing to astrocytic region‐specific transcriptional dependencies.^[^ ^85^, ^86^ ^]^ Moreover, recent studies have identified several astrocyte‐derived signals that can modulate neuronal lipid metabolism and synaptic function. For example, astrocyte‐derived microRNAs (miRNAs) have been shown to regulate the expression of genes involved in neuronal cholesterol synthesis and efflux.^[^ ^87^ ^]^ Additionally, the synaptic vesicle protein Mover, which is secreted by astrocytes, has been implicated in the formation of neuronal LDs and the regulation of synaptic plasticity.^[^ ^88^ ^]^

However, the metabolic coupling between astrocytes and neurons can be disrupted in various neurological disorders and during aging. For instance, in AD, the APOE4 variant impairs the transport of FFAs from neurons to astrocytes and disrupts the catabolism of FFAs in astrocytes,^[^ ^62^, ^68^ ^]^ leading to neuronal lipotoxicity and synaptic dysfunction. Moreover, the accumulation of beta‐amyloid (Aβ) and tau proteins in AD can induce mitochondrial dysfunction and oxidative stress in both astrocytes and neurons, further exacerbating the metabolic imbalance. In aging and schizophrenia, the transcriptional synchrony between astrocytes and neurons declines, which may contribute to the impairment of metabolic coupling and cognitive function.^[^ ^89^ ^]^

In conclusion, the metabolic coupling between astrocytes and neurons is a complex and dynamic process that involves the exchange of energy substrates, lipids, and signaling molecules. LDs in astrocytes play a crucial role in this metabolic crosstalk by regulating lipid metabolism, preventing lipotoxicity, and modulating synaptic function. Targeting the astrocyte–neuron metabolic coupling and LD metabolism may represent a promising therapeutic strategy for various neurological disorders characterized by metabolic dysfunction and synaptic impairment.

#### 4.4.2 Astrocyte–Immune Cell Interactions

Microglia, the resident immune cells of the CNS, play a critical role in regulating neuroinflammation and lipid metabolism. Activated microglia secrete proinflammatory cytokines and lipid mediators that can modulate astrocytic LD metabolism. For example, microglial‐derived 25‐hydroxycholesterol (25‐HC), produced by cholesterol 25‐hydroxylase (CH25H), can inhibit cholesterol synthesis and promote LD formation in astrocytes.^[^ ^90^ ^]^ Moreover, microglia can induce the formation of neurotoxic A1 astrocytes through the secretion of IL‐1α, TNF, and C1q, which can further exacerbate neuroinflammation and neurodegeneration.^[^ ^91^ ^]^ During demyelination, reactive astrocytes can express numerous ligands, including Fractalkine (CX3CL1), colony‐stimulating factor 1 (CSF1), IL‐34, and growth arrest‐specific 6 (GAS6), which act on homeostatic and potentially mediate microglia activation and recruitment as well as enhance their activity.^[^ ^92^ ^]^ Additionally, microglia can induce the transformation of A1/A2 reactive astrocytes via the C‐X‐C chemokine receptor type (CXCR) 7/phosphoinositide 3‐kinase (PI3K)/protein kinase B (Akt) pathway in chronic post‐surgical pain.^[^ ^93^, ^94^ ^]^

**Figure 3:**
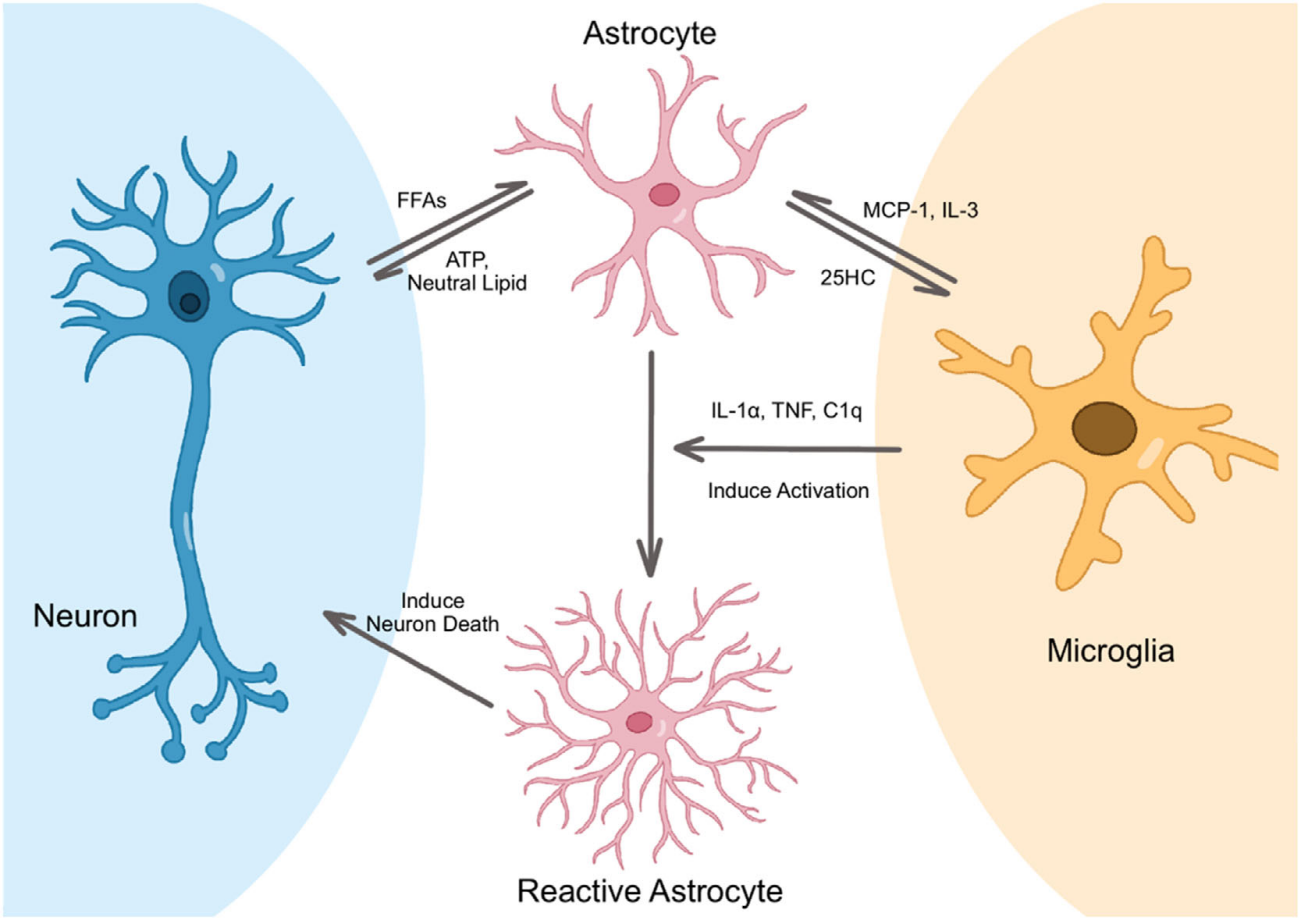
Crosstalk between astrocytes and other nervous system cells related to lipid metabolism. Neurons transfer excess FFAs to astrocytes, which subsequently form LDs and generate ATP and useful neutral lipids. These lipids are then transported back to neurons to support their functions. Microglia highly express cholesterol 25‐hydroxylase, an enzyme that catalyzes the production of 25‐hydroxycholesterol (25HC), which regulates cholesterol metabolism in astrocytes. Astrocytes can activate microglia through the secretion of various factors, such as monocyte chemoattractant protein‐1 (MCP‐1) and interleukin‐3 (IL‐3), leading to increased inflammation. Conversely, microglia can induce the generation of A1 reactive astrocytes by secreting interleukin‐1α (IL‐1α), tumor necrosis factor (TNF), and complement component 1q (C1q). A1 reactive astrocytes gain the ability to induce neuronal death.

LD‐laden astrocytes can also activate microglia through monocyte chemotactic protein 1 (MCP‐1),^[^ ^95^ ^]^ IL‐3,^[^ ^68^ ^]^ and other factors, inducing microglia to migrate towards the inflammatory site and secrete inflammatory mediators, thereby exacerbating neuroinflammation (**Figure** **3**).

#### 4.4.3 Astrocyte–Oligodendrocyte Interactions

Oligodendrocytes, the myelin‐forming cells of the CNS,^[^ ^96^ ^]^ require a large supply of lipids for membrane synthesis and myelin production. Astrocytes support oligodendrocyte function by providing lipids and other metabolic substrates via direct cell‐cell contact as well as secreted cytokines, exosomes, and signaling molecules.^[^ ^97^, ^98^ ^]^ Astrocyte‐derived cholesterol, transported by APOE‐containing lipoproteins, is essential for oligodendrocyte survival and myelination. Moreover, astrocytes can transfer FAs to oligodendrocytes through gap junctions or extracellular vesicles, which can be incorporated into myelin lipids or oxidized for energy production. Besides, astrocytes can provide critical metabolic support of oligodendrocytes through SREBP.^[^ ^99^ ^]^

In the context of demyelinating disorders, such as multiple sclerosis (MS), astrocyte–oligodendrocyte lipid transfer can become impaired, contributing to oligodendrocyte dysfunction and myelin loss. In MS, reactive astrocytes exhibit reduced cholesterol synthesis and APOE expression, leading to decreased lipid support for oligodendrocytes.^[^ ^100^, ^101^ ^]^ Furthermore, the release of proinflammatory cytokines and ROS by reactive astrocytes can induce oxidative damage and apoptosis in oligodendrocytes.^[^ ^102^, ^103^ ^]^ Promoting astrocyte–oligodendrocyte lipid transfer and reducing astrocyte reactivity may represent potential therapeutic strategies for demyelinating disorders.

#### 4.4.4 Astrocyte–Endothelial Cell Interactions

Astrocytes play a crucial role in maintaining BBB integrity and regulating lipid transport across the BBB. Astrocytic end‐feet ensheathe brain capillaries and regulate endothelial cell function through the release of soluble factors and extracellular vesicles.^[^ ^104^ ^]^ Astrocyte‐derived APOE and ABCs are essential for the transport of cholesterol and other lipids across the BBB. Moreover, astrocytes can secrete factors, such as vascular endothelial growth factor, angiopoietin‐1 (Ang‐1), and MCP‐1,^[^ ^96^ ^]^ which promote endothelial cell survival and stabilize BBB integrity.^[^ ^105^, ^106^ ^]^

In the context of neurological disorders, astrocyte–endothelial cell interactions can become dysregulated, leading to BBB breakdown and lipid accumulation in the brain.^[^ ^107^ ^]^ In AD, reactive astrocytes secrete proinflammatory cytokines and ROS that can disrupt BBB integrity and increase the infiltration of peripheral immune cells and lipids into the brain. Besides, the accumulation of Aβ peptides in the brain can induce oxidative damage and dysfunction in both astrocytes and endothelial cells, further exacerbating BBB breakdown and neuroinflammation.^[^ ^108^ ^]^ Moreover, elevation of blood TAGs can induce stress, inflammation, and apoptosis pathways, making BBB endothelial cells experience lipotoxicity, thus causing an increase number of LDs in astrocytes as well as promoting AD progress.^[^ ^109^ ^]^ Under the condition of hemorrhagic stroke, matrix metalloproteinase‐3 (MMP3) was highly upregulated in inflammatory reactive astrocytes, which can disrupt the integrity of BBB.^[^ ^110^, ^111^ ^]^ Targeting astrocyte–endothelial cell interactions and promoting BBB integrity may represent potential therapeutic strategies for neurological disorders associated with BBB dysfunction.

### 4.5 Astrocytic LD Metabolism in Aging

Aging is a major risk factor for neurological disorders, and an aging brain usually features with compromised bioenergetics, impaired neuroplasticity, aberrant neuronal network activity, disorder of neuronal Ca^2+^ homeostasis, inflammation, and peroxidation.^[^ ^112^ ^]^ Accumulating evidence suggests that alterations in astrocytic LD metabolism may contribute to age‐related cognitive decline and disease pathogenesis.^[^ ^56^ ^]^ In the aging brain, astrocytes exhibit increased LD accumulation, which is associated with impaired FA oxidation, mitochondrial dysfunction, and oxidative stress.^[^ ^56^ ^]^ It is noteworthy that moderate LD number seems to have specific benefits to our health,^[^ ^72^ ^]^ and aging seems to be always accompanied by decreased synthesis and increased catabolism of cholesterol.^[^ ^113^ ^]^ Moreover, the expression of LDAPs, such as PLIN2,^[^ ^48^ ^]^ is upregulated in astrocytes during aging, further promoting LD formation and stabilization.

Lots of molecular mechanisms have been implicated in the dysregulation of astrocytic LD metabolism during aging.^[^ ^114^ ^]^ Age‐related changes in lipid metabolism, such as increased cholesterol synthesis and reduced cholesterol efflux, can lead to LD accumulation in astrocytes.^[^ ^78^ ^]^ The expression of lipid transport proteins, including APOE^[^ ^115^ ^]^ and ABCs,^[^ ^116^ ^]^ also declines with age, impairing lipid trafficking and LD turnover. Furthermore, the activity of transcriptional regulators, such as LXRs^[^ ^117^ ^]^ and peroxisome proliferator‐activated receptors (PPARs),^[^ ^118^ ^]^ is reduced in aged astrocytes, resulting in decreased expression of genes involved in FA oxidation and mitochondrial function. Additionally, dysfunction of organelles, like abnormal OxPhos in mitochondrion,^[^ ^68^ ^]^ accumulated cholesterol in lysosomal,^[^ ^119^ ^]^ will also disrupt lipid metabolism homeostasis, promoting aging progress.

Cellular senescence, a hallmark of aging, may also contribute to the dysregulation of astrocytic LD metabolism.^[^ ^120^ ^]^ Senescent astrocytes exhibit increased LD accumulation, which is associated with the senescence‐associated secretory phenotype (SASP), characterized by the release of proinflammatory cytokines and lipid mediators.^[^ ^120^, ^121^ ^]^ The SASP can further exacerbate neuroinflammation and oxidative stress, creating a vicious cycle that promotes LD accumulation and astrocyte dysfunction. Moreover, stress and/or injury signals will trigger a cooperation of microglia and astrocytes, amplifying neuroinflammation and contributing to the release of neurotoxic factors.^[^ ^23^ ^]^

Interestingly, interventions that modulate astrocytic LD metabolism have shown promise in ameliorating age‐related cognitive decline. For example, activation of PPARs^[^ ^122^ ^]^ by natural or synthetic agonists can enhance FA oxidation and mitochondrial function in astrocytes, reducing LD accumulation and improving cognitive performance in aged animals. Similarly, increasing the expression of APOE and ABCs through genetic or pharmacological approaches can promote lipid efflux and LD turnover, attenuating age‐related neuroinflammation and oxidative stress.^[^ ^123^ ^]^

## 5 Techniques of LD Monitoring

Advances in LD research have driven the rapid development of LD detection technology, including the analysis of LD lipid components, LD‐specific labeling, and the observation of LD dynamics in live cells.

### 5.1 Lipidomic Analysis

Lipidomics primarily focuses on investigating the types, distribution, functions, interactions with other biomolecules, and dynamic changes of various lipids in physiological metabolism and pathological states. Mass spectrometry is the most widely used technique in lipidomic analysis,^[^ ^61^ ^]^ further revealing the specific functions of lipids and their metabolic changes in diseases. In the study of LDs, lipidomics concentrates on analyzing the composition of LD components, including physiological states and lipid metabolism changes caused by gene knockout and protein alterations.^[^ ^124^ ^]^ In 2023, Minami and her team integrated lipidomics, transcriptomics, and genomics data from various glioblastoma (GBM) tumor and derivative models to detect the therapeutic exploitation link between recurrent molecular lesions and lipid metabolism changes in GBM.^[^ ^125^ ^]^

### 5.2 Live Cell Imaging Technology

Specific labeling of LDs typically involves the use of specific antibodies and fluorescent dyes. Antibodies targeting LDAPs, such as PLIN2, are often employed to label LDs.^[^ ^56^ ^]^ Currently, the main fluorescent labeling dyes used for LD visualization include BODIPY 493/503,^[^ ^83^ ^]^ Oil Red O solution,^[^ ^52^ ^]^ and Nile Red.^[^ ^126^ ^]^ These dyes are used to stain LDs and enable their quantification under a fluorescence microscope. Oil Red O and Nile Red staining are commonly used for observing LDs in tissue sections, as they bind to lipids and label them red, allowing for the quantitative analysis of LD number and size. The most distinctive characteristic of BODIPY dyes is their asymmetry, which enables the effective conjugation of labeled groups, such as lipophilic structures. By localizing to polar lipids within cells, BODIPY dyes can specifically stain both live and fixed cells.

Due to the lack of LD specificity in the aforementioned staining methods, some researchers have proposed the use of indolyl‐benzothiadiazole derivatives for imaging LD accumulation in astrocytes and GBM cells. By modifying the electronic properties or size of the N‐substituent on the indole motif, the fluorescence characteristics of these compounds can be highly adjustable, avoiding nonspecific staining or background artifacts.^[^ ^127^ ^]^ Moreover, these compounds can penetrate BBB, laying the foundation for subsequent in vivo studies.

### 5.3 Novel Fluorescent Probes

Research on the biological process of LDs has promoted the development of dynamic monitoring technology. Recently, super‐resolution imaging techniques have emerged to decipher physiological processes and molecular mechanisms at the nanoscale. These emerging super‐resolution fluorescence imaging technologies include structured illumination microscopy (SIM), stimulated emission depletion microscopy (STED), and photoactivated localization microscopy (PALM). However, these techniques have additional stringent requirements for fluorescent probes, and currently, the spatial and temporal resolution, as well as long‐term imaging capabilities of fluorescent probes, are still limited.

Some studies suggest that metal‐based probes can provide better imaging of LDs.^[^ ^128^ ^]^ With high LD specificity, these probes can be used to visualize lipid metabolism in living cells and organisms through optical imaging. However, they are easily quenched by oxygen and/or water, resulting in poor long‐term imaging ability. To address this issue, Xu et al. proposed a new concept of buffered fluorescence probes (BFPs) to significantly reduce photobleaching in SIM super‐resolution imaging of LDs and achieve light‐stable long‐term SIM imaging.^[^ ^129^, ^130^ ^]^ Cao et al. synthesized an ultrabright solvatochromic fluorescent probe based on benzoboranils, which possesses ultrahigh fluorescence quantum yields due to its structure rigidification, for use in SIM imaging.^[^ ^131^ ^]^ Dai et al. developed a fluorescent probe, Lipi‐Deep Red, with excellent photostability in the deep red/near‐infrared (NIR) emission range for SIM imaging.^[^ ^132^ ^]^ Zhou et al. introduced a LD fluorescent probe, Lipi‐QA, with ultrastrong photostability and long fluorescence lifetime for STED imaging.^[^ ^133^ ^]^ Dual‐channel fluorescent probes can respond to a specific target and emit fluorescence at different wavelengths before and after the response. Li et al. proposed a NIR fluorescence probe activated by cysteine (Cys), which can simultaneously monitor Cys in mitochondria and LDs during cell apoptosis through wash‐free fluorescence bioimaging. This probe has demonstrated superior efficacy in the in vivo imaging of cell apoptosis in chronic epilepsy mice.^[^ ^134^ ^]^

## 6 Current Studies on LDs in Neurological Diseases

### 6.1 Neurodegenerative Diseases

Abnormal lipid metabolism is considered a crucial factor influencing the progression of neurodegenerative diseases, including AD, Parkinson's disease (PD), Huntington's disease (HD), hereditary spastic paraplegia (HSP), amyotrophic lateral sclerosis (ALS), and other related disorders.^[^ ^3^, ^135^ ^]^

**Figure 4:**
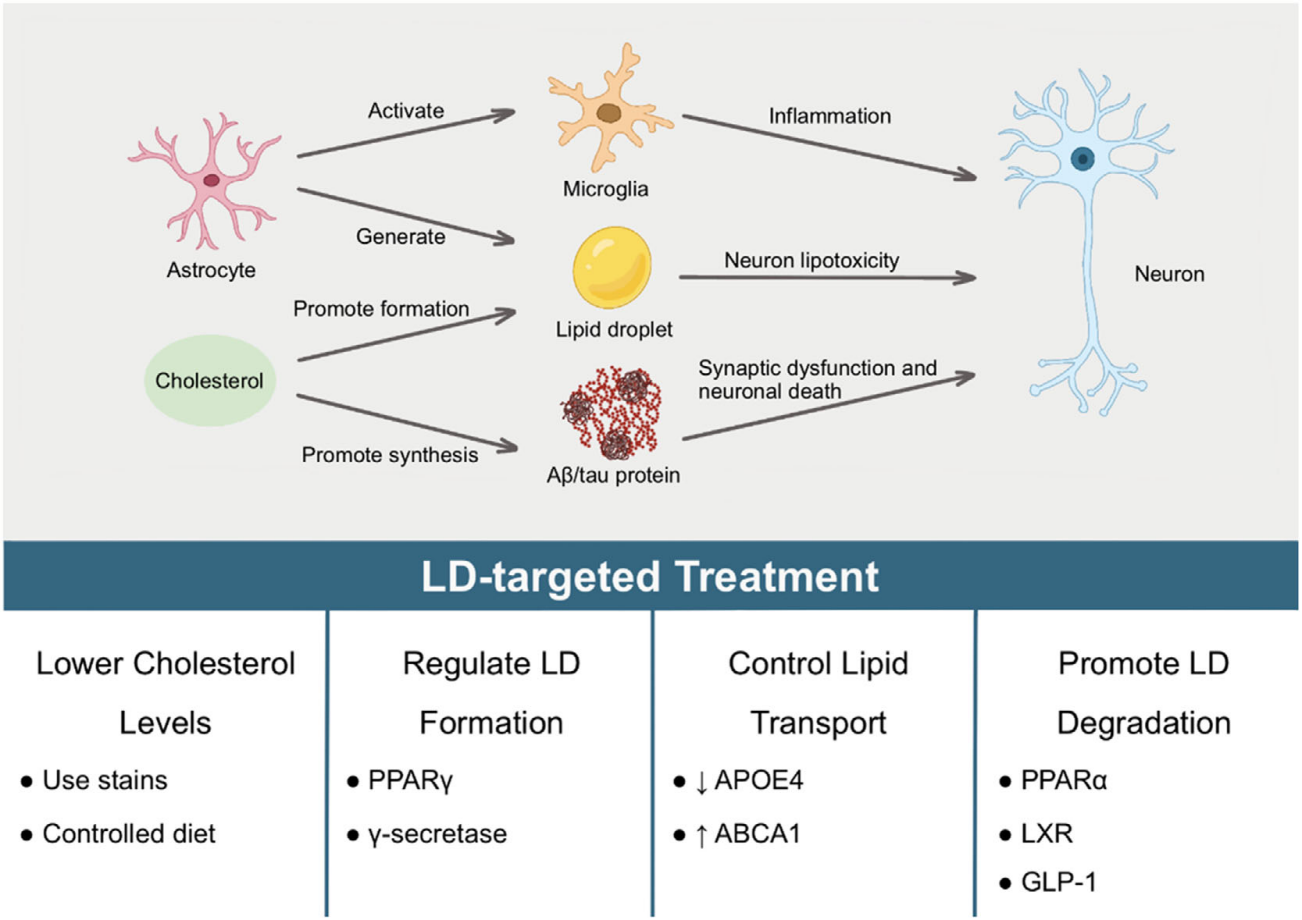
Current research on the mechanisms and clinical applications of LDs in AD. The exploration of LD mechanisms in AD centers on three primary aspects: inflammation, neuronal lipotoxicity associated with LDs, and the synaptic dysfunction and neuronal death related to amyloid‐β (Aβ) and tau proteins. Reactive astrocytes, which harbor elevated levels of unsaturated FAs, can activate microglia, thereby promoting inflammation and exhibiting neuronal lipotoxicity. An overabundance of cholesterol not only facilitates LD formation but also contributes to the accumulation of amyloid‐β (Aβ) and tau proteins, thereby exacerbating AD pathology. Current clinical strategies targeting LD metabolism for the treatment of AD encompass lowering cholesterol levels, regulating LD formation, managing lipid transport, and enhancing LD degradation. Lowering cholesterol levels can be achieved through the use of statins and dietary modifications. Regulation of LD formation involves modulating peroxisome proliferator‐activated receptor (PPAR) γ and γ‐secretase activity. Management of lipid transport includes suppressing apolipoprotein E4 (APOE4) and activating ATP‐binding cassette transporter A1 (ABCA1). Finally, promoting LD degradation can be accomplished through the regulation of PPAR α, as well as utilizing liver X receptor (LXR) agonists and glucagon‐like peptide‐1 (GLP‐1) analogs.

Recent research indicates a significant correlation between cholesterol metabolism and the development of AD (**Figure** **4**). Firstly, excessive brain cholesterol independently regulates the deposition of tau and Aβ, while neuronal cholesterol reduction induces phosphorylated tau degradation by increasing proteasome levels and total cell proteasome activity.^[^ ^136^ ^]^ Tau, a kind of protein assisting in maintaining the stability of the internal structure of neurons under physiological conditions, has been regarded as a crucial factor of helping glial LD formation and protecting against neuronal toxic peroxidated lipids (LPOs). While pathological, tau will occur abnormal phosphorylation thus promoting the production of neurofibrillary tangle (NFT).^[^ ^137^ ^]^ Aβ, a primary factor triggering NFT formation,^[^ ^138^, ^139^ ^]^ can also promote an increase in cytoplasmic Ca^2+^ concentration^[^ ^140^ ^]^ in neurons and induce nuclear factor κB (NF‐κB) activation,^[^ ^141^ ^]^ which will in turn accelerate formation of Aβ oligomers and deteriorate neuroinflammation.^[^ ^142^ ^]^ Another research showed that neuronal amyloid precursor protein (APP) is specifically transported in and out of lipid clusters via astrocyte‐derived cholesterol, where it interacts with β‐ and γ‐secretases to produce Aβ‐peptide.^[^ ^143^ ^]^ Mutations in presenilin (PSEN) 1 and PSEN2 were also causative of early‐onset familial AD, via activating γ‐secretases and regulating lipid metabolism in the CNS.^[^ ^144^ ^]^ Furthermore, LD‐overloaded cells found in aging brains may promote the formation of autophagosomes within cells, increase the phenotype of aging cells, and release TNF‐α to promote inflammation.^[^ ^145^ ^]^ FA‐overloaded astrocytes can also induce the generation of reactive astrocytes, activate microglial inflammatory responses, and cause neuronal death, leading to lipid toxicity and accelerating the progression of AD.^[^ ^68^ ^]^ Reactive astrocytes recently have been regarded as the more and more important factor during AD progress, which can deregulate synaptogenic molecules, thrombospondin (TSP) 1 and TSP2, and glutathione peroxidase (GYP) 4 and GYP6, impact glutamate reuptake and recycling, and increase the release of inflammatory factors.^[^ ^146^ ^]^ And conversely, Tau protein and Aβ can also trigger inflammation pathways, like STAT3, and induce the formation of reactive astrocytes.^[^ ^68^, ^147^ ^]^ Compared to reactive astrocytes, astrocytes can release antioxidant factor like nuclear factor Nrf2, to activate neuroprotective functional changes,^[^ ^148^ ^]^ indicating that enhancing the adaptive‐protective responses of astrocytes may be a good direction to treat AD patients. APOE4 constitutes a significant risk factor for AD development,^[^ ^149^, ^150^ ^]^ contributing to tau formation and disrupting lipid metabolic homeostasis.^[^ ^151^ ^]^ Notably, targeted deletion of astrocytic APOE4 significantly attenuates tau‐induced synaptic loss and microglial phagocytosis of synaptic structures.^[^ ^152^ ^]^ Furthermore, CNS myelin sulfatide deficiency results in APOE upregulation, which subsequently promotes Aβ accumulation, thereby exacerbating neuroinflammatory processes and accelerating age‐related pathologies.^[^ ^153^, ^154^ ^]^ Even in cases of chronic human immunodeficiency virus (HIV) infection, lipid metabolism disorders remain the basis for accelerated brain aging.^[^ ^155^ ^]^

The regulation of lipid metabolism represents a new direction in the treatment of neurodegenerative diseases. Researchers have proposed exploring the regulation or inhibition of γ‐secretase to identify targeted treatments for AD and certain cancers.^[^ ^66^, ^156^ ^]^ Glucagon‐like peptide‐1 receptor agonists (GLP‐1 RAs) are primarily used for cardiovascular diseases and blood glucose control. In the context of AD pathology, GLP‐1 can enhance aerobic glycolysis in glial cells through the PI3K/Akt pathway, thereby promoting FA oxidation, reducing ROS production, and enhancing support for neurons.^[^ ^157^ ^]^

Studies have also found that NLY01, a GLP‐1 RA, exerts neuroprotective effects by directly preventing the transformation of astrocytes into the A1 neurotoxic phenotype mediated by microglia,^[^ ^94^ ^]^ proposing a new direction for controlling the progression of neurodegenerative diseases with hyperglycemia or cardiovascular diseases. Additionally, activating lipid‐sensitive nuclear receptors, including retinoid X receptors (RXRs),^[^ ^158^ ^]^ LXRs, and PPARs,^[^ ^159^ ^]^ inhibiting de novo synthesis of FAs in the brain, promoting lipid transport proteins (APOE and ABCA1), and administering cholesterol‐lowering drugs are also important strategies for treating AD.^[^ ^160^ ^]^

Studies have also found that a high‐fat diet (HFD) can cause disturbances in lipid metabolism in astrocytes, accelerating the progression of neurodegenerative diseases,^[^ ^120^ ^]^ while lipid metabolism disorders, in turn, can exacerbate diet‐induced obesity.^[^ ^161^ ^]^ Notably, diets with differences in FA saturation resulted in minor differences in the brain lipidome, whereas diets enriched in the n‐3FAs, docosahexaenoic acid (DHA) and eicosapentaenoic acid (EPA), can significantly alter the phospholipid FA composition.^[^ ^162^ ^]^ Therefore, the potential mechanisms and treatments of neurodegenerative diseases related to various dietary interventions have become popular research topics.^[^ ^163^ ^]^ Conjugated linoleic acid can decrease the levels of TNF‐α, IL‐1β, and CCL5 in cultured human astrocytes, thereby regulating neuroinflammation.^[^ ^164^ ^]^ A ketogenic diet helps the brain utilize ketone bodies generated by the liver while upregulating the biogenesis of neuronal mitochondria, improving brain energy supply, and slowing down the progression of AD.^[^ ^165^ ^]^ Besides, recently, researchers also found dietary supplementation of mono‐unsaturated FAs (MUFA) can upregulate LDs, reduce lipid oxidation, and extend lifespan.^[^ ^72^ ^]^

### 6.2 Epilepsy

Traditionally, research on epilepsy has focused on glucose metabolism and disturbances in potassium metabolism. Seizure activity has a high energy demand to maintain neuronal overactivity, which ultimately leads to severe cellular and mitochondrial dysregulation.^[^ ^166^ ^]^ In recent years, studies have discovered that LDs accumulate at the site of epileptic lesions, and the disappearance of LD fluorescence can be observed after treatment with the antiepileptic drug curcumin,^[^ ^134^ ^]^ revealing that lipid metabolism disturbances can influence seizure activity. Chen et al. found that lipid‐accumulated reactive astrocytes (LARAs) can promote the progression of epilepsy, and intervention in neuronal‐astrocyte lipid transfer or metabolism can reduce the formation of LARAs and alleviate seizure activity. In epilepsy, LARAs appear to promote neuronal discharge and disease progression by inducing abnormal lipid accumulation within the epileptic lesion and upregulating adenosine 2a receptor (A2AR).^[^ ^4^ ^]^ Building on this foundation, Yang et al. proposed that mitochondrial dysfunction may play a crucial role in the pathogenesis of temporal lobe epilepsy.^[^ ^167^ ^]^ However, a substantial amount of experimental evidence is still needed to elucidate the related mechanisms, and treatments aimed at regulating lipid metabolism to prevent seizures are also future research directions.

### 6.3 IS

Lipid metabolism dysfunction is widely involved in the pathological process of IS. However, most studies suggest that microglia are the primary cause of lipid metabolism disorders during IS.^[^ ^168^ ^]^ Loannou et al. found that astrocytes accumulate LDs after acute stroke, mainly related to neuroinflammation.^[^ ^40^ ^]^ Using lipidomics analysis, Huang et al. discovered that long‐chain acylcarnitine (LCAC) can serve as a novel and promising diagnostic and prognostic biomarker for acute IS (AIS).^[^ ^5^ ^]^ LCAC can accumulate and damage neurons by inducing mitochondrial dysfunction in astrocytes in AIS. Reducing LCAC or stimulating astrocyte FA oxidation (FAO) can ameliorate stroke injury. Oxygen‐glucose deprivation/reperfusion (OGD/R) stimulates neurons to transfer FFAs to astrocytes, leading to LD formation. These LDs can be released into mitochondria through lipolysis to produce LCACs, resulting in mitochondrial dysfunction.

Regulating lipid metabolism disorders and controlling neuroinflammation are promising directions for the treatment of IS. In 2021, Wei et al. proposed that de novo adipogenesis in astrocytes promotes BBB repair after transient cerebral ischemia through IL‐33.^[^ ^169^ ^]^ In 2024, they also discovered that glycerol triacetate can improve BBB repair and functional recovery after IS by enhancing lipogenesis and IL‐33 expression. They suggested that exogenous IL‐33 can also improve BBB repair and long‐term functional recovery after stroke,^[^ ^170^ ^]^ which is a potential future treatment approach. Guan et al. proposed a novel hybrid of telmisartan and borneol to reduce neuroinflammation and white matter damage in IS through the activating transcription factor 3 (ATF3)/CH25H axis.^[^ ^171^ ^]^ Guo et al. found that after OGD/R stimulation, seipin deficiency exacerbates neuroinflammation by activating the toll‐like receptor 3 (TLR3)/tumor necrosis factor receptor‐associated factors (TRAF3)/NF‐κB signaling pathway, suggesting that seipin may be a potential therapeutic target for AIS.^[^ ^172^ ^]^ Ferroptosis, as an oxidative stress regulator, has also been found to play a role in lipid metabolism disorders in IS. Targeting ferroptosis as a way to alleviate cell damage and death is another potential direction for treatment.^[^ ^173^ ^]^

### 6.4 Glioma

LDs and ferroptosis are emerging targets for studying the progression and treatment resistance of gliomas.^[^ ^174^ ^]^ Astrocytes regulate the recruitment of tumor‐associated macrophages (TAMs) to the tumor microenvironment (TME) through CCL2 and support the tumor phenotype by promoting TAMs, partially through the release of macrophage colony‐stimulating factor. Glioma cells also rely on cholesterol derived from astrocytes for survival,^[^ ^6^ ^]^ indicating that depleting tumor‐associated astrocytes (TAAs) or targeting cholesterol efflux in TAAs can inhibit tumor development, thereby prolonging survival time. Minami et al. developed a large library of molecularly annotated GBM tumors and derivative in vitro and in vivo orthotopic models. They found that cyclin‐dependent kinase inhibitor 2 A (CDKN2A) deficiency reshapes lipid metabolism, leading to ferroptosis in GBM, highlighting the research value of ferroptosis in GBM.^[^ ^125^ ^]^

The conventional treatment of gliomas generally involves surgery, and in cases where surgery is not feasible, other treatments such as radiotherapy or chemotherapy need to be considered. Recent studies have revealed that regulating lipid metabolism, such as SOAT1,^[^ ^175^ ^]^ may reduce resistance to radiotherapy/chemotherapy, thereby increasing the survival rate of glioma patients.^[^ ^176^ ^]^ Ancient ubiquitous protein 1 (AUP1) is believed to regulate LD catabolism and has recently been identified as a poor predictive biomarker associated with tumor proliferation, reflecting neuroinflammatory conditions.^[^ ^177^ ^]^ The unmethylated promoter region of O6‐methylguanine‐DNA methyltransferase (MGMT) is an important biomarker for identifying chemo‐resistant subtypes of GBM. Hao et al. discovered that MGMT methylation can also serve as a specific biomarker to classify lipid metabolism patterns between methylated and unmethylated GBM^[^ ^178^ ^]^ and proposed the potential development of atorvastatin in the treatment of GBM with unmethylated MGMT.^[^ ^179^ ^]^ De Martino et al. found that radiation therapy can promote unsaturated FAs, creating a suitable lipid growth environment for GBM and driving GBM resistance. They proposed that targeted lipid metabolism may improve the efficiency of radiation therapy for GBM.^[^ ^180^ ^]^ More details can be found in **Table** **2**.

**Table 2:** Current studies on LDs in neurological diseases. ABCA1: triphosphate‐binding cassette transporter 1; AD: Alzheimer's disease; ALS: amyotrophic lateral sclerosis; APOE: apolipoprotein E; ATF3: activating transcription factor 3; CDKN2A: cyclin‐dependent kinase inhibitor 2a; CH25H: cholesterol 25 hydroxylase; DGAT: diacylglycerol *O*‐acyltransferase; FABP: FA binding protein; GBM: glioblastoma; GLP‐1: glucagon‐like peptide‐1; IL: interleukin; iPSC: induced pluripotent stem cells; IS: ischemic stroke; LD: lipid droplets; LDLR: low density lipoprotein receptor; NF‐κB: nuclear factor κB; PD: Parkinson disease; PPAR: peroxisome proliferator‐activated receptor; SOAT1: sterol *O*‐acyltransferase 1; SREBP: sterol regulatory element‐binding protein; TLR3: toll‐like receptor 3; TNF: tumor necrosis factor; TRAF3: tumor necrosis factor receptor‐associated factors 3.

Diseases	Publish year	Study type	Key findings	Reference
AD	2018	In vitro study	APOE4 causes widespread molecular and cellular alterations associated with AD phenotypes in Human iPSC‐derived brain cell types.	[182]
AD	2020	Clinical trial	PPAR δ/γ agonist showed dose‐dependent systemic effects on lipid metabolism and metabolism related to insulin sensitization in AD patients.	[183]
AD	2020	Clinical trial	Medium‐chain TAGs improved cognition and lipid metabolomics in mild to moderate AD patients with APOE4.	[184]
AD	2022	In vivo study	Gut microbiota modulation can alter lipid composition thus slow down AD progress.	[185]
AD	2023	In vitro and in vivo study	Chronic suppression of γ‐secretase impairs synapses by lowering cholesterol levels.	[20]
AD	2023	In vitro study	Amyloid‐β accumulation induces mitochondrial disruption and altered energy metabolism.	[141]
AD	2023	In vitro and in vivo study	The acetate‐boosted reactive astrocyte–neuron interaction could contribute to the cognitive decline in AD.	[186]
AD	2024	in vivo study	FABP7 drives an inflammatory response in human astrocytes and is upregulated in AD.	[187]
AD	2024	In vivo study	Tau is required for glial LD formation and resistance to neuronal oxidative stress.	[136]
AD	2024	In silico study	This article used a data‐driven approach and reconstructed the dynamics of the brain's cellular environment and identified a trajectory leading to AD.	[188]
PD	2018	In vivo study	GLP‐1 agonist can block A1 neurotoxic astrocyte conversion by microglia in models of PD.	[93]
PD	2022	In vivo study	Linoleic acid has neuroprotective and anti‐inflammatory benefit through regulating LD dynamics in PD patients.	[189]
PD	2018	Clinical trial	Omega‐3 FAs and vitamin E co‐supplementation can improve gene expression of TNF‐α, PPAR‐γ and LDLR.	[190]
ALS	2024	In vitro and in vivo study	Pyruvate dehydrogenase kinase 2 knockdown helps astrocytes to support motor neuron survival in ALS mice.	[191]
Neurodegenerative Disease	2021	Review	Cholesterol metabolism in neurodegenerative diseases	[192]
Neurodegenerative Disease	2022	Review	Lipid metabolism and storage in neuroglia of patients with neurodegenerative diseases	[193]
Epilepsy	2018	Review	Astrocytic energy homeostasis is crucial in the control of neuronal excitability as well as treat epilepsy.	[194]
Epilepsy	2022	Review	Effects of astrocytes in the initiation and progression of epilepsy	[195]
Epilepsy	2023	Review	Mitochondrial dysfunction of astrocytes mediates lipid accumulation in temporal lobe epilepsy.	[166]
Epilepsy	2023	In vivo study	Lipid‐accumulated reactive astrocytes promote epilepsy progression.	[4]
IS	2022	In vivo study	Ginsenoside Rb1 can inhibit astrocyte activation and promote transfer of astrocytic mitochondria to neurons against IS	[196]
IS	2023	In vivo study	Glyceryl triacetate promotes BBB recovery after IS through lipogenesis‐mediated IL‐33 in mice.	[169]
IS	2023	In vivo study	Complement C3a treatment accelerates recovery after IS via modulation of astrocyte reactivity and cortical connectivity.	[197]
IS	2023	In vivo study	A robust inflammatory gene signature in brain endothelial cells after IS.	[198]
IS	2024	In vivo study	Lipidomic analysis identifies long‐chain acylcarnitine as a target for IS.	[5]
IS	2024	In vivo study	Hybrid of Telmisartan and Borneol ameliorates neuroinflammation in IS through ATF3/CH25H axis.	[170]
IS	2024	In vivo study	Seipin is involved in oxygen‐glucose deprivation/reoxygenation induced neuroinflammation by regulating the TLR3/TRAF3/NF‐κB pathway.	[171]
Astrocytic Glioma	2022	In vitro study	SOAT1 can be a suitable target for therapy in high‐grade astrocytic glioma.	[41]
GBM	2016	In vivo study and clinical trail	Inhibition of SOAT1 can suppresses glioblastoma growth via blocking SREBP‐1‐mediated lipogenesis.	[199]
GBM	2020	In vitro and in vivo study	Targeting DGAT1 can increase fat catabolism and oxidative stress, thus ameliorates GBM.	[200]
GBM	2022	Review	LDs and ferroptosis are new players in brain cancer GBM progression and therapeutic resistance.	[173]
GBM	2022	In vivo study	Astrocyte‐derived cholesterol is key to glioma cell survival, and that targeting astrocytic cholesterol efflux, via ABCA1, halts tumour progression.	[6]
GBM	2023	Clinical trial	Radiation therapy drives GBM resistance by generating a lipogenic environment permissive to GBM survival.	[179]
GBM	2023	In vitro and in vivo study, patient tumor analysis	CDKN2A deletion remodels lipid metabolism to prime GBM for ferroptosis.	[124]

## 7 Therapeutic Implications and Future Directions

Given the crucial role of astrocytic LD metabolism in the pathogenesis of neurological disorders, targeting LD‐associated pathways represents a promising therapeutic strategy. In this section, we discuss the potential therapeutic implications and future directions of research on astrocytic LD metabolism, focusing on clinical applications, drug delivery, and key research questions.

### 7.1 Clinical Applications and Biomarker Potential

Accumulating evidence suggests that LDs and their lipid components have the potential to serve as disease biomarkers in clinical settings, reflecting the development, progression, and treatment response of neurological disorders. Firstly, LDs exhibit cell type and brain region‐specific distribution patterns, with higher abundance in glial cells compared to neurons. The quantity and size of LDs in astrocytes can reflect the level of lipid metabolism and indicate the degree of neuroinflammation and disease progression. For example, a recent study using a BODIPY‐based fluorescent probe demonstrated that targeted imaging of astrocytic LDs can facilitate the early diagnosis of AD by detecting Aβ aggregates and altered LD viscosity.^[^ ^181^ ^]^

Secondly, the lipid composition of LDs can provide disease‐specific information. Lipidomic analyses have identified several lipid species enriched in LDs that are associated with specific neurological disorders. For instance, long‐chain acylcarnitine have been proposed as potential biomarkers for IS,^[^ ^5^ ^]^ while the methylation status of the MGMT promoter has been linked to distinct lipid metabolism patterns in glioblastoma.^[^ ^179^ ^]^ Moreover, the expression of LDAPs, such as AUP1,^[^ ^177^ ^]^ has been correlated with glioblastoma proliferation and neuroinflammation.

Thirdly, LD dynamics and distribution exhibit sensitivity to the progression of neuroinflammation and aging.^[^ ^182^ ^]^ Quantitative imaging and lipidomic approaches have revealed that the number and size of LDs in astrocytes increase in response to inflammatory stimuli and during the aging process. Furthermore, LD analysis has shown good reproducibility and reliability, with acceptable interindividual variations.

These findings highlight the potential of astrocytic LDs as novel biomarkers for the diagnosis, prognosis, and treatment monitoring of neurological disorders. However, further research is needed to validate these biomarkers in large‐scale clinical studies and to develop standardized methods for their detection and quantification.

### 7.2 Therapeutic Targeting Strategies

The targeting of astrocytic LD metabolism for therapeutic purposes can be approached from four main perspectives. Firstly, directly modulating the enzymes and proteins involved in LD biogenesis and catabolism can regulate LD accumulation and lipid homeostasis. For example, inhibiting DGAT enzymes or activating ATGL can reduce LD formation and promote the mobilization of stored lipids, respectively. Secondly, targeting the regulatory factors of LD metabolism, such as APOE, neuronal activity, γ‐secretase, and adrenergic signaling, can indirectly influence LD dynamics and lipid trafficking.

Thirdly, modulating the interactions between LDs and other organelles, such as mitochondria and lysosomes, can impact LD metabolism and cellular energetics. This can be achieved by targeting the proteins that mediate organelle contacts, such as PLIN5 and ORP8, or by regulating the motility and distribution of LDs within the cell. Fourthly, targeting the intercellular transport of LDs and their lipid cargo between astrocytes and other neural cell types can modulate the availability and distribution of lipids in the brain. This can be accomplished by targeting the proteins involved in lipid transfer, such as APOE and ABCA1, or by modulating the secretion and uptake of extracellular vesicles containing LDs.

In addition to these direct targeting approaches, modulating neuroinflammation and oxidative stress can indirectly influence astrocytic LD metabolism. Neuroinflammatory mediators, such as cytokines and reactive oxygen species, have been shown to stimulate LD accumulation in astrocytes. Therefore, anti‐inflammatory and antioxidant therapies may have beneficial effects on LD metabolism and lipid homeostasis in the brain.

### 7.3 Drug Delivery Considerations

Effective therapeutic targeting of astrocytic LD metabolism requires the development of drug delivery systems that can cross the BBB and specifically reach the desired brain regions and cell types. Nanoparticle‐based drug delivery platforms, such as liposomes, polymeric nanoparticles, and exosomes, have shown promise in enhancing the brain delivery of LD‐targeting drugs. These nanocarriers can be engineered to display astrocyte‐specific targeting ligands, such as glutamate transporter inhibitors or connexin hemichannel blockers, to improve their selectivity and reduce off‐target effects.

Another approach to enhance drug delivery to the brain is to exploit the endogenous transport systems at the BBB, such as receptor‐mediated transcytosis and adsorptive‐mediated transcytosis. For example, the use of APOE‐mimetic peptides or antibodies against the transferrin receptor has been shown to facilitate the transport of therapeutic agents across the BBB. Furthermore, the intranasal route of administration has emerged as a noninvasive and efficient method for delivering drugs directly to the brain, bypassing the BBB.

In addition to drug delivery, the development of noninvasive imaging techniques for monitoring LD metabolism and drug response in vivo is crucial for the successful translation of LD‐targeting therapies. Advanced imaging modalities, such as MRS, PET, and Raman spectroscopy, have the potential to provide quantitative and spatiotemporal information on lipid distribution and metabolism in the living brain.

### 7.4 Future Research Directions

While significant progress has been made in understanding the role of astrocytic LD metabolism in neurological disorders, several key questions remain to be addressed. Firstly, the precise molecular mechanisms underlying the dysregulation of LD metabolism in astrocytes and its contribution to neuroinflammation, oxidative stress, and neurodegeneration need to be further elucidated. This will require the integration of multiomics approaches, including lipidomics, proteomics, and metabolomics, to gain a systems‐level understanding of the complex network of factors regulating LD metabolism in health and disease.

Secondly, future investigations should leverage spatial transcriptomics and single‐cell lipidomics technologies to elucidate the molecular heterogeneity of astrocytic lipid metabolism across different brain regions, disease stages, and patient populations. By simultaneously resolving spatial distribution and molecular signatures at single‐cell resolution, these advanced approaches can reveal distinct metabolic profiles of astrocyte subpopulations and their differential contributions to disease pathogenesis. Integration of these high‐dimensional datasets with clinical parameters may uncover novel biomarkers and therapeutic targets specific to particular astrocyte populations, enabling more precise diagnostic and treatment strategies for neurological disorders characterized by lipid metabolic dysregulation.

Thirdly, the crosstalk between astrocytic LD metabolism and other glial cell types, such as microglia and oligodendrocytes, in the context of neurological disorders warrants further investigation. Understanding the complex interplay between these cells in regulating lipid homeostasis and neuroinflammation may provide new insights into disease mechanisms and therapeutic opportunities.

Fourthly, the translational potential of targeting astrocytic LD metabolism should be rigorously evaluated in relevant preclinical models and clinical trials. This will require the development of standardized protocols for drug formulation, delivery, and dosing, as well as the identification of appropriate patient populations and clinical endpoints. Furthermore, the safety and long‐term efficacy of LD‐targeting therapies should be carefully assessed, given the critical role of lipid metabolism in brain function and development.

Finally, the development of innovative imaging technologies and probes for visualizing and quantifying LD dynamics and lipid distribution in the brain will be essential for advancing our understanding of LD metabolism in neurological disorders and for guiding therapeutic interventions. The combination of super‐resolution microscopy, microspectroscopy, and mass spectrometry imaging techniques may provide unprecedented insights into the spatial and temporal organization of LDs and their interactions with other cellular components at the nanoscale level.

## 8 Conclusion

Astrocytes play a central role in brain lipid metabolism, and the dysregulation of astrocytic LD metabolism has emerged as a key contributor to the pathogenesis of neurological disorders. LDs are dynamic organelles that regulate lipid storage, trafficking, and signaling in astrocytes, and their accumulation is associated with neuroinflammation, oxidative stress, and neurodegeneration. This review has provided a comprehensive overview of the current knowledge on astrocytic LD biology and its implications in neurological disorders.

We have discussed the structural and functional heterogeneity of astrocytes, the molecular mechanisms regulating LD metabolism, and the crosstalk between astrocytes and other neural cell types in lipid homeostasis. We have highlighted the emerging role of astrocytic LDs as potential biomarkers and therapeutic targets in neurodegenerative diseases, stroke, epilepsy, and glioma. Furthermore, we have outlined the key challenges and future directions in translating these findings into clinical applications, including drug delivery, biomarker development, and imaging technologies.

In conclusion, the study of astrocytic LD metabolism represents a promising new frontier in neurological research, with important implications for understanding disease mechanisms and developing targeted therapies. By integrating multidisciplinary approaches and leveraging advanced analytical tools, future research in this field may lead to the identification of novel diagnostic and prognostic biomarkers, as well as the development of innovative lipid‐based therapies for neurological disorders. Ultimately, a deeper understanding of the complex interplay between astrocytes, lipid metabolism, and brain function may provide new insights into the fundamental biology of the CNS and its dysregulation in disease states.

## Conflict of Interest

The authors declare no conflict of interest.

## Author Contributions

**Jiani Zhong**: data curation, methodology, writing—original draft, validation. **Yanyi Peng**: visualization, editing. **Lingyue Zhang**: methodology **Bo Xiao**: resources, project administration; **Mengqi Zhang**: conceptualization, writing—review and editing, supervision, funding acquisition, project administration.

## Consent for Publication

All authors have agreed to publish.

## References

[1] H. G. Lee , M. A. Wheeler , F. J. Quintana , Nat. Rev. Drug Discovery 2022, 21, 339. 35173313 10.1038/s41573-022-00390-x PMC9081171

[2] W. Sun , Z. Liu , X. Jiang , M. B. Chen , H. Dong , J. Liu , T. C. Südhof , S. R. Quake , Nature 2024, 627, 374. 38326616 10.1038/s41586-023-07011-6 PMC10937396

[3] B. C. Farmer , A. E. Walsh , J. C. Kluemper , L. A. Johnson , Front. Neurosci. 2020, 14, 742. 32848541 10.3389/fnins.2020.00742 PMC7403481

[4] Z. P. Chen , S. Wang , X. Zhao , W. Fang , Z. Wang , H. Ye , M. J. Wang , L. Ke , T. Huang , P. Lv , X. Jiang , Q. Zhang , L. Li , S. T. Xie , J. N. Zhu , C. Huang , D. Chen , X. Liu , C. Yan , Nat. Neurosci., 2023, 26, 542. 36941428 10.1038/s41593-023-01288-6

[5] X. X. Huang , L. Li , R. H. Jiang , J. B. Yu , Y. Q. Sun , J. Shan , J. Yang , J. Ji , S. Q. Cheng , Y. F. Dong , X. Y. Zhang , H. B. Shi , S. Liu , X. L. Sun , J. Adv. Res. 2023, 5, 6.

[6] R. Perelroizen , B. Philosof , N. Budick-Harmelin , T. Chernobylsky , A. Ron , R. Katzir , D. Shimon , A. Tessler , O. Adir , A. Gaoni-Yogev , T. Meyer , A. Krivitsky , N. Shidlovsky , A. Madi , E. Ruppin , L. Mayo , Brain 2022, 145, 3288. 35899587 10.1093/brain/awac222 PMC10233310

[7] J. A. Olzmann , P. Carvalho , Nat. Rev. Mol. Cell Biol. 2019, 20, 137. 30523332 10.1038/s41580-018-0085-z PMC6746329

[8] M. Majchrzak , O. Stojanović , D. Ajjaji , K. Ben M'barek , M. Omrane , A. R. Thiam , R. W. Klemm , Cell Rep. 2024, 43, 114093. 38602875 10.1016/j.celrep.2024.114093

[9] A. J. Mathiowetz , J. A. Olzmann , Nat. Cell Biol. 2024, 26, 331. 38454048 10.1038/s41556-024-01364-4 PMC11228001

[10] T. C. Walther , J. Chung , R. V. Farese , Ann. Rev. Cell Dev. Biol. 2017, 33, 491. 28793795 10.1146/annurev-cellbio-100616-060608 PMC6986389

[11] M. Majchrzak , O. Stojanović , D. Ajjaji , K. Ben M'barek , M. Omrane , A. R. Thiam , R. W. Klemm , Science 2022, 378, eadc9020. 38602875 10.1016/j.celrep.2024.114093

[12] B. Zhou , Y. X. Zuo , R. T. Jiang , CNS Neurosci. Ther. 2019, 25, 665. 30929313 10.1111/cns.13123 PMC6515705

[13] W. P. Li , X. H. Su , N. Y. Hu , J. Hu , X. W. Li , J. M. Yang , T. M. Gao , Biol Psychiatry. 2022, 92, 984. 35787318 10.1016/j.biopsych.2022.04.019

[14] M. I. Adam , L. Lin , A. M. Makin , X. F. Zhang , L. X. Zhou , X. Y. Liao , L. Zhao , F. Wang , D. S. Luo , Neural Regener. Res. 2023, 18, 1364. 10.4103/1673-5374.354517 PMC9838158 36453424

[15] J. V. Andersen , A. Schousboe , A. Verkhratsky , Prog. Neurobiol. 2022, 217, 102331. 35872221 10.1016/j.pneurobio.2022.102331

[16] C. X. Tan , C. J. Burrus Lane , C. Eroglu , Curr. Top Dev. Biol. 2021, 142, 371. 33706922 10.1016/bs.ctdb.2020.12.010

[17] Y. Liu , X. Shen , Y. Zhang , X. Zheng , C. Cepeda , Y. Wang , S. Duan , X. Tong , Glia 2023, 71, 1383. 36799296 10.1002/glia.24343

[18] Y. Xie , A. T. Kuan , W. Wang , Z. T. Herbert , O. Mosto , O. Olukoya , M. Adam , S. Vu , M. Kim , D. Tran , N. Gómez , C. Charpentier , I. Sorour , T. E. Lacey , M. Y. Tolstorukov , B. L. Sabatini , W. A. Lee , C. C. Harwell , Cell Rep. 2022, 38, 110416. 35196485 10.1016/j.celrep.2022.110416 PMC8962654

[19] A. F. van Deijk , N. Camargo , J. Timmerman , T. Heistek , J. F. Brouwers , F. Mogavero , H. D. Mansvelder , A. B. Smit , M. H. Verheijen , Glia 2017, 65, 670. 28168742 10.1002/glia.23120

[20] S. Essayan‐Perez , T. C. Südhof , Neuron 2023, 111, 3176. 37543038 10.1016/j.neuron.2023.07.005 PMC10592349

[21] S. Takahashi , Cells 2022, 11, 813.35269435

[22] A. Mishra , J. Physiol. 2017, 595, 1885. 27619153 10.1113/JP270979 PMC5350479

[23] A. Picca , E. Ferri , R. Calvani , H. J. Coelho‐Júnior , E. Marzetti , B. Arosio , Nutrients 2022, 14, 2406. 35745134 10.3390/nu14122406 PMC9230668

[24] A. Eraso‐Pichot , S. Pouvreau , A. Olivera‐Pinto , P. Gomez‐Sotres , U. Skupio , G. Marsicano , Glia 2023, 71, 44. 35822691 10.1002/glia.24246 PMC9796923

[25] M. Velasco‐Estevez , S. O. Rolle , M. Mampay , K. K. Dev , G. K. Sheridan , Glia 2020, 68, 145. 31433095 10.1002/glia.23709

[26] Y. M. Zhang , Y. B. Qi , Y. N. Gao , W. G. Chen , T. Zhou , Y. Zang , J. Li , Front. Neurosci. 2023, 17, 1217451. 37732313 10.3389/fnins.2023.1217451 PMC10507181

[27] D. Singh , J. Neuroinflammation 2022, 19, 206. 35978311 10.1186/s12974-022-02565-0 PMC9382837

[28] T. Wang , Q. Sun , J. Yang , G. Wang , F. Zhao , Y. Chen , Y. Jin , Food Chem. Toxicol. 2021, 157, 112550. 34517076 10.1016/j.fct.2021.112550

[29] B. He , L. Niu , S. Li , H. Li , Y. Hou , A. Li , X. Zhang , H. Hao , H. Song , R. Cai , Y. Zhou , Y. Wang , Y. Wang , Brain Behav. Immun. 2024, 116, 85. 38042209 10.1016/j.bbi.2023.11.035

[30] J. M. Lawrence , K. Schardien , B. Wigdahl , M. R. Nonnemacher , Acta Neuropathol. Commun. 2023, 11, 42. 36915214 10.1186/s40478-023-01526-9 PMC10009953

[31] S. Köhler , U. Winkler , J. Hirrlinger , Neurochem. Res. 2021, 46, 3. 31797158 10.1007/s11064-019-02926-x

[32] C. Escartin , E. Galea , A. Lakatos , J. P. O’Callaghan , G. C. Petzold , A. Serrano-Pozo , C. Steinhäuser , A. Volterra , G. Carmignoto , A. Agarwal , N. J. Allen , A. Araque , L. Barbeito , A. Barzilai , D. E. Bergles , G. Bonvento , A. M. Butt , W. T. Chen , M. Cohen-Salmon , C. Cunningham , B. Deneen , B. De Strooper , B. Díaz-Castro , C. Farina , M. Freeman , V. Gallo , J. E. Goldman , S. A. Goldman , M. Götz , A. Gutiérrez , et al., Nat. Neurosci. 2021, 24, 312. 33589835 10.1038/s41593-020-00783-4 PMC8007081

[33] E. G. Cameron , M. Nahmou , A. B. Toth , L. Heo , B. Tanasa , R. Dalal , W. Yan , P. Nallagatla , X. Xia , S. Hay , C. Knasel , T. L. Stiles , C. Douglas , M. Atkins , C. Sun , M. Ashouri , M. Bian , K. C. Chang , K. Russano , S. Shah , M. B. Woodworth , J. Galvao , R. V. Nair , M. S. Kapiloff , J. L. Goldberg , Nature 2024, 626, 574. 38086421 10.1038/s41586-023-06935-3 PMC11384621

[34] Y. Y. Fan , J. Huo , Neurochem. Int. 2021, 148, 105080. 34048845 10.1016/j.neuint.2021.105080

[35] L. E. Clarke , S. A. Liddelow , C. Chakraborty , A. E. Münch , M. Heiman , B. A. Barres , Proc. Natl. Acad. Sci. 2018, 115, E1896. 29437957 10.1073/pnas.1800165115 PMC5828643

[36] J. Chang , Z. Qian , B. Wang , J. Cao , S. Zhang , F. Jiang , R. Kong , X. Yu , X. Cao , L. Yang , H. Chen , Cell Commun. Signal 2023, 21, 37. 36797790 10.1186/s12964-022-01036-6 PMC9936716

[37] Z. Qian , J. Qin , Y. Lai , C. Zhang , X. Zhang , Biomolecules 2023, 13, 692. 37189441 10.3390/biom13040692 PMC10135484

[38] S. Feng , C. Wu , P. Zou , Q. Deng , Z. Chen , M. Li , L. Zhu , F. Li , T. C. Liu , R. Duan , L. Yang , Theranostics 2023, 13, 3434. 37351177 10.7150/thno.81951 PMC10283053

[39] R. V. Farese Jr , T. C. Walther , Cell 2009, 139, 855. 19945371 10.1016/j.cell.2009.11.005 PMC3097139

[40] M. S. Ioannou , J. Jackson , S. H. Sheu , C. L. Chang , A. V. Weigel , H. Liu , H. A. Pasolli , C. S. Xu , S. Pang , D. Matthies , H. F. Hess , J. Lippincott-Schwartz , Z. Liu , Cell 2019, 177, 1522. 31130380 10.1016/j.cell.2019.04.001

[41] M. Löhr , W. Härtig , A. Schulze , M. Kroiß , S. Sbiera , C. Lapa , B. Mages , S. Strobel , J. E. Hundt , S. Bohnert , S. Kircher , S. Janaki-Raman , C. M. Monoranu , Int. J. Mol. Sci. 2022, 23, 3726. 35409086 10.3390/ijms23073726 PMC8998855

[42] N. Mejhert , K. R. Gabriel , S. Frendo-Cumbo , N. Krahmer , J. Song , L. Kuruvilla , C. Chitraju , S. Boland , D. K. Jang , M. von Grotthuss , M. C. Costanzo , M. Rydén , J. A. Olzmann , J. Flannick , N. P. Burtt , R. V. Farese Jr. , T. C. Walther , Dev Cell 2022, 57, 387. 35134345 10.1016/j.devcel.2022.01.003 PMC9129885

[43] C. Sztalryd , D. L. Brasaemle , Biochim. Biophys. Acta Mol. Cell Biol. Lipids 2017, 1862, 1221. 28754637 10.1016/j.bbalip.2017.07.009 PMC5595658

[44] J. G. Granneman , V. A. Kimler , H. Zhang , X. Ye , X. Luo , J. H. Postlethwait , R. Thummel , eLife 2017, 6, e21771. 28244868 10.7554/eLife.21771 PMC5342826

[45] P. Chandrasekaran , S. Weiskirchen , R. Weiskirchen , J. Cell Biochem. 2024, 125, e30579. 38747370 10.1002/jcb.30579

[46] M. A. Roberts , K. K. Deol , A. J. Mathiowetz , M. Lange , D. E. Leto , J. Stevenson , S. H. Hashemi , D. W. Morgens , E. Easter , K. Heydari , M. A. Nalls , M. C. Bassik , M. Kampmann , R. R. Kopito , F. Faghri , J. A. Olzmann , Dev. Cell 2023, 58, 1782. 37494933 10.1016/j.devcel.2023.07.001 PMC10530302

[47] E. Griseti , A. A. Bello , E. Bieth , B. Sabbagh , J. S. Iacovoni , J. Bigay , H. Laurell , A. Čopič , FEBS Lett. 2023, 598, 1170. 10.1002/1873-3468.14792 38140813

[48] M. Conte , V. Medici , D. Malagoli , A. Chiariello , A. Cirrincione , A. Davin , M. Chikhladze , F. Vasuri , G. Legname , I. Ferrer , S. Vanni , G. Marcon , T. E. Poloni , A. Guaita , C. Franceschi , S. Salvioli , Neuropathol. App.l Neurobiol. 2022, 48, e12756. 10.1111/nan.12756 PMC9291275 34312912

[49] S. Guo , L. Hou , L. Dong , X. Nie , L. Kang , X. Wang , Nat. Ecol. Evol. 2023, 7, 914. 37156891 10.1038/s41559-023-02059-z

[50] R. V. Farese Jr , T. C. Walther , Cold Spring Harbor Perspect. Biol. 2023, 15, a041246. 10.1101/cshperspect.a041246 PMC10153804 36096640

[51] R. J. Schulze , A. Sathyanarayan , D. G. Mashek , Biochim. Biophys. Acta Mol. Cell Biol. Lipids 2017, 1862, 1178. 28642194 10.1016/j.bbalip.2017.06.008 PMC5595645

[52] M. B. Schott , S. G. Weller , R. J. Schulze , E. W. Krueger , K. Drizyte-Miller , C. A. Casey , M. A. McNiven , J. Cell Biol. 2019, 218, 3320. 31391210 10.1083/jcb.201803153 PMC6781454

[53] M. Pu , W. Zheng , H. Zhang , W. Wan , C. Peng , X. Chen , X. Liu , Z. Xu , T. Zhou , Q. Sun , D. Neculai , W. Liu , Protein Cell 2023, 14, 653. 37707322 10.1093/procel/pwac063 PMC10501187

[54] N. Shatz , Y. Chohan , D. J. Klionsky , Autophagy 2024, 20, 1697. 38735055 10.1080/15548627.2024.2350739 PMC11262226

[55] G. F. Grabner , H. Xie , M. Schweiger , R. Zechner , Nat. Metab. 2021, 3, 1445. 34799702 10.1038/s42255-021-00493-6

[56] T. Smolič , P. Tavčar , A. Horvat , U. Černe , A. Halužan Vasle , L. Tratnjek , M. E. Kreft , N. Scholz , M. Matis , T. Petan , R. Zorec , N. Vardjan , Glia 2021, 69, 1540. 33609060 10.1002/glia.23978 PMC8248329

[57] A. Horvat , R. Zorec , N. Vardjan , Front. Physiol. 2021, 12, 735532. 34658920 10.3389/fphys.2021.735532 PMC8514727

[58] I. A. Windham , S. Cohen , Trends Cell Biol. 2024, 34, 338. 37805344 10.1016/j.tcb.2023.09.004 PMC10995109

[59] G. Qi , Y. Mi , X. Shi , H. Gu , R. D. Brinton , F. Yin , Cell Rep. 2021, 34, 108572. 33406436 10.1016/j.celrep.2020.108572 PMC7837265

[60] I. A. Windham , A. E. Powers , J. V. Ragusa , E. D. Wallace , M. C. Zanellati , V. H. Williams , C. H. Wagner , K. K. White , S. Cohen , J. Cell Biol. 2024, 223, e202305003. 38334983 10.1083/jcb.202305003 PMC10857907

[61] J. Tcw , L. Qian , N. H. Pipalia , M. J. Chao , S. A. Liang , Y. Shi , B. R. Jain , S. E. Bertelsen , M. Kapoor , E. Marcora , E. Sikora , E. J. Andrews , A. C. Martini , C. M. Karch , E. Head , D. M. Holtzman , B. Zhang , M. Wang , F. R. Maxfield , W. W. Poon , A. M. Goate , Cell 2022, 185, 2213. 35750033 10.1016/j.cell.2022.05.017 PMC9340815

[62] G. Sienski , P. Narayan , J. M. Bonner , N. Kory , S. Boland , A. A. Arczewska , W. T. Ralvenius , L. Akay , E. Lockshin , L. He , B. Milo , A. Graziosi , V. Baru , C. A. Lewis , M. Kellis , D. M. Sabatini , L. H. Tsai , S. Lindquist , Sci. Transl. Med. 2021, 13, eaaz4564. 33658354 10.1126/scitranslmed.aaz4564 PMC8218593

[63] K. A. Guttenplan , M. K. Weigel , P. Prakash , P. R. Wijewardhane , P. Hasel , U. Rufen-Blanchette , A. E. Münch , J. A. Blum , J. Fine , M. C. Neal , K. D. Bruce , A. D. Gitler , G. Chopra , S. A. Liddelow , B. A. Barres , Nature 2021, 599, 102. 34616039 10.1038/s41586-021-03960-y PMC12054010

[64] R. Zorec , N. Vardjan , Neurobiol. Dis. 2023, 182, 106132. 37094775 10.1016/j.nbd.2023.106132

[65] L. Pardo , L. M. Valor , A. Eraso-Pichot , A. Barco , A. Golbano , G. E. Hardingham , R. Masgrau , E. Galea , Sci. Rep. 2017, 7, 6390. 28743894 10.1038/s41598-017-06231-x PMC5526874

[66] E. Gutierrez , D. Lütjohann , A. Kerksiek , M. Fabiano , N. Oikawa , L. Kuerschner , C. Thiele , J. Walter , Life Sci. Alliance 2020, 3, e201900521. 32354700 10.26508/lsa.201900521 PMC7195048

[67] A. M. Valm , S. Cohen , W. R. Legant , J. Melunis , U. Hershberg , E. Wait , A. R. Cohen , M. W. Davidson , E. Betzig , J. Lippincott-Schwartz , Nature 2017, 546, 162. 28538724 10.1038/nature22369 PMC5536967

[68] Y. Mi , G. Qi , F. Vitali , Y. Shang , A. C. Raikes , T. Wang , Y. Jin , R. D. Brinton , H. Gu , F. Yin , Nat. Metab. 2023, 5, 445. 36959514 10.1038/s42255-023-00756-4 PMC10202034

[69] I. Y. Benador , M. Veliova , M. Liesa , O. S. Shirihai , Cell Metab. 2019, 29, 827. 30905670 10.1016/j.cmet.2019.02.011 PMC6476311

[70] D. Yang , X. Wang , L. Zhang , Y. Fang , Q. Zheng , X. Liu , W. Yu , S. Chen , J. Ying , F. Hua , Cell Biosci. 2022, 12, 106. 35831869 10.1186/s13578-022-00828-0 PMC9277953

[71] I. Y. Benador , M. Veliova , K. Mahdaviani , A. Petcherski , J. D. Wikstrom , E. A. Assali , R. Acín-Pérez , M. Shum , M. F. Oliveira , S. Cinti , C. Sztalryd , W. D. Barshop , J. A. Wohlschlegel , B. E. Corkey , M. Liesa , O. S. Shirihai , Cell Metab. 2018, 27, 869. 29617645 10.1016/j.cmet.2018.03.003 PMC5969538

[72] K. Papsdorf , J. W. Miklas , A. Hosseini , M. Cabruja , C. S. Morrow , M. Savini , Y. Yu , C. G. Silva-García , N. R. Haseley , L. M. Murphy , P. Yao , E. de Launoit , S. J. Dixon , M. P. Snyder , M. C. Wang , W. B. Mair , A. Brunet , Nat. Cell Biol. 2023, 25, 672. 37127715 10.1038/s41556-023-01136-6 PMC10185472

[73] J. S. Soto , Y. Jami-Alahmadi , J. Chacon , S. L. Moye , B. Diaz-Castro , J. A. Wohlschlegel , B. S. Khakh , Nature 2023, 616, 764. 37046092 10.1038/s41586-023-05927-7 PMC10132990

[74] G. Bonvento , J. P. Bolaños , Cell Metab. 2021, 33, 1546. 34348099 10.1016/j.cmet.2021.07.006

[75] A. Almeida , D. Jimenez‐Blasco , J. P. Bolaños , Essays Biochem. 2023, 67, 17. 36805653 10.1042/EBC20220075 PMC10011404

[76] A. Suzuki , S. A. Stern , O. Bozdagi , G. W. Huntley , R. H. Walker , P. J. Magistretti , C. M. Alberini , Cell 2011, 144, 810. 21376239 10.1016/j.cell.2011.02.018 PMC3073831

[77] J. M. Medina , A. Tabernero , J. Neurosci. Res. 2005, 79, 2. 15573408 10.1002/jnr.20336

[78] F. W. Pfrieger , N. Ungerer , Prog. Lipid Res. 2011, 50, 357. 21741992 10.1016/j.plipres.2011.06.002

[79] T. Smolič , R. Zorec , N. Vardjan , Antioxidants 2021, 11, 22. 35052526 10.3390/antiox11010022 PMC8773017

[80] L. Chen , X. W. Chen , X. Huang , B. L. Song , Y. Wang , Y. Wang , Sci. China Life Sci. 2019, 62, 1420. 31686320 10.1007/s11427-019-1563-3

[81] L. Liu , K. R. MacKenzie , N. Putluri , M. Maletić‐Savatić , H. J. Bellen , Cell Metab. 2017, 26, 719. 28965825 10.1016/j.cmet.2017.08.024 PMC5677551

[82] T. B. Nguyen , S. M. Louie , J. R. Daniele , Q. Tran , A. Dillin , R. Zoncu , D. K. Nomura , J. A. Olzmann , Dev. Cell 2017, 42, 9. 28697336 10.1016/j.devcel.2017.06.003 PMC5553613

[83] S. Datta , M. Jaiswal , Mitochondrion 2021, 59, 135. 33895346 10.1016/j.mito.2021.04.006

[84] L. Groc , D. Choquet , Science 2020, 368, eaay4631. 32527803 10.1126/science.aay4631

[85] Y. T. Cheng , E. Luna-Figueroa , J. Woo , H. C. Chen , Z. F. Lee , A. S. Harmanci , B. Deneen , Nature 2023, 617, 369. 37100909 10.1038/s41586-023-06010-x PMC10733939

[86] J. V. Andersen , A. Schousboe , P. Wellendorph , Essays Biochem. 2023, 67, 77. 36806927 10.1042/EBC20220208

[87] X. Li , J. Zhang , D. Li , C. He , K. He , T. Xue , L. Wan , C. Zhang , Q. Liu , Neuron 2021, 109, 957. 33503410 10.1016/j.neuron.2021.01.005

[88] J. Krohn , F. Domart , T. T. Do , T. Dresbach , Glia 2023, 71, 2799. 37539560 10.1002/glia.24452

[89] E. Ling , J. Nemesh , M. Goldman , N. Kamitaki , N. Reed , R. E. Handsaker , G. Genovese , J. S. Vogelgsang , S. Gerges , S. Kashin , S. Ghosh , J. M. Esposito , K. Morris , D. Meyer , A. Lutservitz , C. D. Mullally , A. Wysoker , L. Spina , A. Neumann , M. Hogan , K. Ichihara , S. Berretta , S. A. McCarroll , Nature 2024, 627, 604. 38448582 10.1038/s41586-024-07109-5 PMC10954558

[90] A. G. Cashikar , D. Toral-Rios , D. Timm , J. Romero , M. Strickland , J. M. Long , X. Han , D. M. Holtzman , S. M. Paul , S. A. Liddelow , K. A. Guttenplan , L. E. Clarke , F. C. Bennett , C. J. Bohlen , L. Schirmer , M. L. Bennett , A. E. Münch , W. S. Chung , T. C. Peterson , D. K. Wilton , A. Frouin , B. A. Napier , N. Panicker , M. Kumar , M. S. Buckwalter , D. H. Rowitch , V. L. Dawson , T. M. Dawson , B. Stevens , B. A. Barres , J. Lipid Res. 2023, 64, 100350. 36849076 10.1016/j.jlr.2023.100350 PMC10060115

[91] F. C. Bennett , C. J. Bohlen , L. Schirmer , M. L. Bennett , A. E. Münch , W. S. Chung , T. C. Peterson , D. K. Wilton , A. Frouin , B. A. Napier , N. Panicker , M. Kumar , M. S. Buckwalter , D. H. Rowitch , V. L. Dawson , B. Stevens , B. A. Barres , Nature 2017, 541, 481. 28099414 10.1038/nature21029 PMC5404890

[92] L. J. Schröder , F. Mulenge , A. Pavlou , T. Skripuletz , M. Stangel , V. Gudi , U. Kalinke , Glia 2023, 71, 2573. 37455566 10.1002/glia.24440

[93] T. Li , T. Liu , X. Chen , L. Li , M. Feng , Y. Zhang , L. Wan , C. Zhang , W. Yao , J. Neuroinflammation 2020, 17, 211. 32665021 10.1186/s12974-020-01891-5 PMC7362409

[94] S. P. Yun , T. I. Kam , N. Panicker , S. Kim , Y. Oh , J. S. Park , S. H. Kwon , Y. J. Park , S. S. Karuppagounder , H. Park , S. Kim , N. Oh , N. A. Kim , S. Lee , S. Brahmachari , X. Mao , J. H. Lee , M. Kumar , D. An , S. U. Kang , Y. Lee , K. C. Lee , D. H. Na , D. Kim , S. H. Lee , V. V. Roschke , S. A. Liddelow , Z. Mari , B. A. Barres , V. L. Dawson , Nat. Med. 2018, 24, 931. 29892066 10.1038/s41591-018-0051-5 PMC6039259

[95] Y. H. Kwon , J. Kim , C. S. Kim , T. H. Tu , M. S. Kim , K. Suk , D. H. Kim , B. J. Lee , H. S. Choi , T. Park , M. S. Choi , T. Goto , T. Kawada , T. Y. Ha , R. Yu , FEBS Lett. 2017, 591, 1742. 28542876 10.1002/1873-3468.12691

[96] D. M. McTigue , R. B. Tripathi , J. Neurochem. 2008, 107, 1. 18643793 10.1111/j.1471-4159.2008.05570.x

[97] E. Nutma , D. van Gent , S. Amor , L. A. N. Peferoen , Cells 2020, 9, 600. 32138223 10.3390/cells9030600 PMC7140446

[98] J. Li , L. Zhang , Y. Chu , M. Namaka , B. Deng , J. Kong , X. Bi , Front. Cell Neurosci. 2016, 10, 119. 27242432 10.3389/fncel.2016.00119 PMC4861901

[99] N. Camargo , A. Goudriaan , A. F. van Deijk , W. M. Otte , J. F. Brouwers , H. Lodder , D. H. Gutmann , K. A. Nave , R. M. Dijkhuizen , H. D. Mansvelder , R. Chrast , A. B. Smit , M. H. G. Verheijen , PLoS Biol. 2017, 15, e1002605. 28549068 10.1371/journal.pbio.1002605 PMC5446120

[100] I. Molina-Gonzalez , R. K. Holloway , Z. Jiwaji , O. Dando , S. A. Kent , K. Emelianova , A. F. Lloyd , L. H. Forbes , A. Mahmood , T. Skripuletz , V. Gudi , J. A. Febery , J. A. Johnson , J. H. Fowler , T. Kuhlmann , A. Williams , S. Chandran , M. Stangel , A. J. M. Howden , G. E. Hardingham , V. E. Miron , Nat. Commun. 2023, 14, 3372. 37291151 10.1038/s41467-023-39046-8 PMC10250470

[101] K. K. Mok , S. H. Yeung , G. W. Cheng , I. W. Ma , R. H. Lee , K. Herrup , K. H. TseI , W. Ma , R. H. Lee , K. Herrup , K. H. Tse , J. Neurochem. 2023, 165, 55. 36549843 10.1111/jnc.15748

[102] J. Zheng , J. Lu , S. Mei , H. Wu , Z. Sun , Y. Fang , S. Xu , X. Wang , L. Shi , W. Xu , S. Chen , J. Yu , F. Liang , J. Zhang , J. Neuroinflammation 2021, 18, 43. 33588866 10.1186/s12974-021-02101-6 PMC7883579

[103] Z. Su , Y. Yuan , J. Chen , Y. Zhu , Y. Qiu , F. Zhu , A. Huang , C. He , J. Neurotrauma 2011, 28, 1089. 21309692 10.1089/neu.2010.1597

[104] M. D. Sweeney , K. Kisler , A. Montagne , A. W. Toga , B. V. Zlokovic , Nat. Neurosci. 2018, 21, 1318. 30250261 10.1038/s41593-018-0234-x PMC6198802

[105] Z. Zhao , A. R. Nelson , C. Betsholtz , B. V. Zlokovic , Cell 2015, 163, 1064. 26590417 10.1016/j.cell.2015.10.067 PMC4655822

[106] N. J. Abbott , L. Rönnbäck , E. Hansson , Nat. Rev. Neurosci. 2006, 7, 41. 16371949 10.1038/nrn1824

[107] Q. Ma , Z. Zhao , A. P. Sagare , Y. Wu , M. Wang , N. C. Owens , P. B. Verghese , J. Herz , D. M. Holtzman , B. V. Zlokovic , Mol. Neurodegener. 2018, 13, 57. 30340601 10.1186/s13024-018-0286-0 PMC6194676

[108] R. J. Jackson , J. C. Meltzer , H. Nguyen , C. Commins , R. E. Bennett , E. Hudry , B. T. Hyman , Brain 2022, 145, 3582. 34957486 10.1093/brain/awab478 PMC9586546

[109] L. L. Lee , H. H. Aung , D. W. Wilson , S. E. Anderson , J. C. Rutledge , J. M. Rutkowsky , Am. J. Physiol. Cell Physiol. 2017, 312, C500. 28077357 10.1152/ajpcell.00120.2016 PMC5407020

[110] C. Liu , Y. Guo , S. Deng , S. Zhou , S. Wu , T. Chen , X. Shi , M. Mamtilahun , T. Xu , Z. Liu , H. Li , Z. Zhang , H. Tian , W. S. Chung , J. Wang , G. Y. Yang , Y. Tang , J. Cereb. Blood Flow Metab. 2024, 44, 1102. 38388375 10.1177/0271678X241235008 PMC11179611

[111] D. Feng , J. Zhou , H. Liu , X. Wu , F. Li , J. Zhao , Y. Zhang , L. Wang , M. Chao , Q. Wang , H. Qin , S. Ge , Q. Liu , J. Zhang , Y. Qu , Sci. Adv. 2022, 8, eabq2423. 36179025 10.1126/sciadv.abq2423 PMC9524825

[112] M. P. Mattson , T. V. Arumugam , Cell Metab. 2018, 27. 1176. 29874566 10.1016/j.cmet.2018.05.011 PMC6039826

[113] M. Söderberg , C. Edlund , K. Kristensson , G. Dallner , J. Neurochem. 1990, 54, 415. 2299344 10.1111/j.1471-4159.1990.tb01889.x

[114] A. S. Mutlu , J. Duffy , M. C. Wang , Dev. Cell. 2021, 56, 1394. 33891896 10.1016/j.devcel.2021.03.034 PMC8173711

[115] A. Chemparathy , Y. Le Guen , S. Chen , E. G. Lee , L. Leong , J. E. Gorzynski , T. D. Jensen , A. Ferrasse , G. Xu , H. Xiang , M. E. Belloy , N. Kasireddy , A. Peña-Tauber , K. Williams , I. Stewart , L. Talozzi , T. S. Wingo , J. J. Lah , S. Jayadev , C. M. Hales , E. Peskind , D. D. Child , S. Roeber , C. D. Keene , L. Cong , E. A. Ashley , C. E. Yu , M. D. Greicius , Neuron 2024, 112, 1110. 38301647 10.1016/j.neuron.2024.01.008 PMC10994769

[116] T. Paseban , M. S. Alavi , L. Etemad , A. Roohbakhsh , Expert Opin. Ther. Targets 2023, 27, 531. 37428709 10.1080/14728222.2023.2235718

[117] B. Wang , P. Tontonoz , Nat. Rev. Endocrinol. 2018, 14, 452. 29904174 10.1038/s41574-018-0037-x PMC6433546

[118] H. T. Huang , C. K. Liao , W. T. Chiu , S. F. Tzeng , Int. J. Biochem. Cell Biol. 2017, 86, 42. 28323206 10.1016/j.biocel.2017.03.008

[119] L. G. Allende , L. Natalí , A. B. Cragnolini , M. Bollo , M. M. Musri , D. de Mendoza , M. G. Martín , Glia 2024, 72, 1746. 38856177 10.1002/glia.24580

[120] M. Ogrodnik , Y. Zhu , L. G. P. Langhi , T. Tchkonia , P. Krüger , E. Fielder , S. Victorelli , R. A. Ruswhandi , N. Giorgadze , T. Pirtskhalava , O. Podgorni , G. Enikolopov , K. O. Johnson , M. Xu , C. Inman , A. K. Palmer , M. Schafer , M. Weigl , Y. Ikeno , T. C. Burns , J. F. Passos , T. von Zglinicki , J. L. Kirkland , D. Jurk , Cell Metab. 2019, 29, 1061. 30612898 10.1016/j.cmet.2018.12.008 PMC6509403

[121] T. J. Bussian , A. Aziz , C. F. Meyer , B. L. Swenson , J. M. van Deursen , D. J. Baker , Nature 2018, 562, 578. 30232451 10.1038/s41586-018-0543-y PMC6206507

[122] N. Wang , Y. Zhao , M. Wu , N. Li , C. Yan , H. Guo , Q. Li , Q. Li , Q. Wang , Mol. Neurobiol. 2024, 61, 1187. 37697219 10.1007/s12035-023-03589-0

[123] A. Serrano‐Pozo , S. Das , B. T. Hyman , Lancet Neurol. 2021, 20, 68. 33340485 10.1016/S1474-4422(20)30412-9 PMC8096522

[124] Y. Wu , K. Chen , L. Li , Z. Hao , T. Wang , Y. Liu , G. Xing , Z. Liu , H. Li , H. Yuan , J. Lu , C. Zhang , J. Zhang , D. Zhao , J. Wang , J. Nie , D. Ye , G. Pan , W. Y. Chan , X. Liu , Cell Death Differ. 2022, 29, 2316. 35614132 10.1038/s41418-022-01018-8 PMC9613632

[125] J. K. Minami , D. Morrow , N. A. Bayley , E. G. Fernandez , J. J. Salinas , C. Tse , H. Zhu , B. Su , R. Plawat , A. Jones , A. Sammarco , L. M. Liau , T. G. Graeber , K. J. Williams , T. F. Cloughesy , S. J. Dixon , S. J. Bensinger , D. A. Nathanson , Cancer Cell 2023, 41, 1048. 37236196 10.1016/j.ccell.2023.05.001 PMC10330677

[126] D. Wang , M. Zhang , J. Xu , J. Yang , Molecules 2023, 28, 3121.37049884

[127] K. Colas , K. O. Holmberg , L. Chiang , S. Doloczki , F. J. Swartling , C. Dyrager , RSC Adv. 2021, 11, 23960. 35479010 10.1039/d1ra04419b PMC9036785

[128] B. J. Schwehr , D. Hartnell , M. Massi , M. J. Hackett , Top Curr. Chem. 2022, 380, 46. 10.1007/s41061-022-00400-x PMC9385838 35976575

[129] J. Chen , C. Wang , W. Liu , Q. Qiao , H. Qi , W. Zhou , N. Xu , J. Li , H. Piao , D. Tan , X. Liu , Z. Xu , Angew. Chem. Int. Ed. Engl. 2021, 60, 25104. 34519394 10.1002/anie.202111052

[130] N. Xu , Q. Qiao , X. Fang , G. Wang , K. An , W. Jiang , J. Li , Z. Xu , Anal. Chem. 2024, 96, 4709. 38457637 10.1021/acs.analchem.4c00292

[131] M. Cao , T. Zhu , M. Zhao , F. Meng , Z. Liu , J. Wang , G. Niu , X. Yu , Anal. Chem. 2022, 94, 10676. 35853217 10.1021/acs.analchem.2c00964

[132] J. Dai , Z. Wu , D. Li , G. Peng , G. Liu , R. Zhou , C. Wang , X. Yan , F. Liu , P. Sun , J. Zhou , G. Lu , Biosens. Bioelectron. 2023, 229, 115243. 36989580 10.1016/j.bios.2023.115243

[133] R. Zhou , C. Wang , X. Liang , F. Liu , P. Sun , X. Yan , X. Jia , X. Liu , Y. Wang , G. Lu , Theranostics 2023, 13, 95. 36593956 10.7150/thno.79052 PMC9800742

[134] S. Li , P. Wang , M. Ye , K. Yang , D. Cheng , Z. Mao , L. He , Z. Liu , Anal. Chem. 2023, 95, 5133. 36893258 10.1021/acs.analchem.3c00226

[135] Z. Chen , Z. Yuan , S. Yang , Y. Zhu , M. Xue , J. Zhang , L. Leng , CNS Neurosci. Ther. 2023, 29, 24. 36193573 10.1111/cns.13982 PMC9804080

[136] R. van der Kant , V. F. Langness , C. M Herrera , D. A. Williams , L. K. Fong , Y. Leestemaker , E. Steenvoorden , K. D. Rynearson , J. F. Brouwers , J. B. Helms , H. Ovaa , M. Giera , S. L. Wagner , A. G. Bang , L. S. B. Goldstein , Cell Stem Cell 2019, 24, 363. 30686764 10.1016/j.stem.2018.12.013 PMC6414424

[137] L. D. Goodman , I. Ralhan , X. Li , S. Lu , M. J. Moulton , Y. J. Park , P. Zhao , O. Kanca , Z. S. Ghaderpour Taleghani , J. Jacquemyn , J. M. Shulman , K. Ando , K. Sun , M. S. Ioannou , H. J. Bellen , Nat. Neurosci. 2024, 27, 1918. 39187706 10.1038/s41593-024-01740-1 PMC11809452

[138] J. A. Hardy , G. A. Higgins , Science 1992, 256, 184. 1566067 10.1126/science.1566067

[139] I. Ferrer , Brain Pathol. 2023, 33, e13122. 36223647 10.1111/bpa.13122 PMC9836379

[140] A. Demuro , I. Parker , G. E. Stutzmann , J. Biol. Chem. 2010, 285, 12463. 20212036 10.1074/jbc.R109.080895 PMC2857063

[141] R. E. González‐Reyes , M. O. Nava‐Mesa , K. Vargas‐Sánchez , D. Ariza‐Salamanca , L. Mora‐Muñoz , Front. Mol. Neurosci. 2017, 10, 427. 29311817 10.3389/fnmol.2017.00427 PMC5742194

[142] M. Zyśk , C. Beretta , L. Naia , A. Dakhel , L. Påvénius , H. Brismar , M. Lindskog , M. Ankarcrona , A. Erlandsson , J. Neuroinflammation. 2023, 20, 43. 36803838 10.1186/s12974-023-02722-z PMC9940442

[143] H. Wang , J. A. Kulas , C. Wang , D. M. Holtzman , H. A. Ferris , S. B. Hansen , Proc. Natl. Acad. Sci. 2021, 118, 1918. 10.1073/pnas.2102191118 PMC8379952 34385305

[144] Y. Sun , S. Islam , M. Michikawa , K. Zou , Int. J. Mol. Sci. 2024, 25, 1757. 38339035 10.3390/ijms25031757 PMC10855926

[145] M. K. Shimabukuro , L. G. Langhi , I. Cordeiro , J. M. Brito , C. M. Batista , M. P. Mattson , V. Mello Coelho , Sci. Rep. 2016, 6, 23795. 27029648 10.1038/srep23795 PMC4814830

[146] R. Patani , G. E. Hardingham , S. A. Liddelow , Nat. Rev. Neurol. 2023, 19, 395. 37308616 10.1038/s41582-023-00822-1

[147] Z. Jiwaji , S. S. Tiwari , R. X. Avilés-Reyes , M. Hooley , D. Hampton , M. Torvell , D. A. Johnson , J. McQueen , P. Baxter , K. Sabari-Sankar , J. Qiu , X. He , J. Fowler , J. Febery , J. Gregory , J. Rose , J. Tulloch , J. Loan , D. Story , K. McDade , A. M. Smith , P. Greer , M. Ball , P. C. Kind , P. M. Matthews , C. Smith , O. Dando , T. L. Spires-Jones , J. A. Johnson , S. Chandran , Nat. Commun. 2022, 13, 135. 35013236 10.1038/s41467-021-27702-w PMC8748982

[148] P. Xian , Y. Hei , R. Wang , T. Wang , J. Yang , J. Li , Z. Di , Z. Liu , A. Baskys , W. Liu , S. Wu , Q. Long , Theranostics 2019, 9, 5956. 31534531 10.7150/thno.33872 PMC6735367

[149] Y. Yamazaki , N. Zhao , T. R. Caulfield , C. C. Liu , G. Bu , Nat. Rev. Neurol. 2019, 15, 501. 31367008 10.1038/s41582-019-0228-7 PMC7055192

[150] B. C. Farmer , J. Kluemper , L. A. Johnson , Cells 2019, 8, 182. 30791549 10.3390/cells8020182 PMC6406677

[151] S. Huang , Z. Zhang , J. Cao , Y. Yu , G. Pei , Signal Transduction Target Ther. 2022, 7, 176. 10.1038/s41392-022-01006-x PMC9189105 35691989

[152] C. Wang , M. Xiong , M. Gratuze , X. Bao , Y. Shi , P. S. Andhey , M. Manis , C. Schroeder , Z. Yin , C. Madore , O. Butovsky , M. Artyomov , J. D. Ulrich , D. M. Holtzman , Neuron 2021, 109, 1657. 33831349 10.1016/j.neuron.2021.03.024 PMC8141024

[153] S. Qiu , J. P. Palavicini , J. Wang , N. S. Gonzalez , S. He , E. Dustin , C. Zou , L. Ding , A. Bhattacharjee , C. E. Van Skike , V. Galvan , J. L. Dupree , X. Han , Mol Neurodegener. 2021, 16, 64. 34526055 10.1186/s13024-021-00488-7 PMC8442347

[154] S. Qiu , S. He , J. Wang , H. Wang , A. Bhattacharjee , X. Li , M. Saeed , J. L. Dupree , X. Han , Int. J. Mol. Sci. 2023, 24, 10483. 37445661 10.3390/ijms241310483 PMC10341976

[155] M. L. Guo , Y. Cheng , D. M. Pineda , R. E. Dempsey , L. Yang , Aging Dis. 2024, 16, 454. 38377024 10.14336/AD.2024.0125 PMC11745453

[156] J. A. M. Merilahti , K. Elenius , Oncogene 2019, 38, 151. 30166589 10.1038/s41388-018-0465-z PMC6756091

[157] K. Timper , A. Del Río-Martín , A. L. Cremer , S. Bremser , J. Alber , P. Giavalisco , L. Varela , C. Heilinger , H. Nolte , A. Trifunovic , T. L. Horvath , P. Kloppenburg , H. Backes , J. C. Brüning , Cell Metab. 2020, 31, 1189. 32433922 10.1016/j.cmet.2020.05.001 PMC7272126

[158] Y. Liu , P. Wang , G. Jin , P. Shi, Y. Zhao, J. Guo, Y. Yin, Q. Shao, P. Li, P. Yang, Ageing Res. Rev. 2023, 90, 102021. 37495118 10.1016/j.arr.2023.102021

[159] R. Luo , L. Y. Su , G. Li , J. Yang , Q. Liu , L. X. Yang , D. F. Zhang , H. Zhou , M. Xu , Y. Fan , J. Li , Y. G. Yao , Autophagy 2020, 16, 52. 30898012 10.1080/15548627.2019.1596488 PMC6984507

[160] F. Yin , Febs J. 2023, 290, 1420. 34997690 10.1111/febs.16344 PMC9259766

[161] Y. Gao , C. Layritz , B. Legutko , T. O. Eichmann , E. Laperrousaz , V. S. Moullé , C. Cruciani-Guglielmacci , C. Magnan , S. Luquet , S. C. Woods , R. H. Eckel , C. X. Yi , C. Garcia-Caceres , M. H. Tschöp , Diabetes 2017, 66, 2555. 28710138 10.2337/db16-1278 PMC6463752

[162] D. Fitzner , J. M. Bader , H. Penkert , C. G. Bergner , M. Su , M. T. Weil , M. A. Surma , M. Mann , C. Klose , M. Simons , Cell Rep. 2020, 32, 108132. 32937123 10.1016/j.celrep.2020.108132

[163] Y. L. Xiao , Y. Gong , Y. J. Qi , Z. M. Shao , Y. Z. Jiang , Signal Transduction Target Ther. 2024, 9, 59. 10.1038/s41392-024-01771-x PMC10925609 38462638

[164] F. Saba , A. Sirigu , R. Pillai , P. Caria , L. Cordeddu , G. Carta , E. Murru , V. Sogos , S. Banni , Nutr. Neurosci. 2019, 22, 207. 28847225 10.1080/1028415X.2017.1367130

[165] M. C. L. Phillips , L. M. Deprez , G. M. N. Mortimer , D. K. J. Murtagh , S. McCoy , R. Mylchreest , L. J. Gilbertson , K. M. Clark , P. V. Simpson , E. J. McManus , J. E. Oh , S. Yadavaraj , V. M. King , A. Pillai , B. Romero-Ferrando , M. Brinkhuis , B. M. Copeland , S. Samad , S. Liao , J. A. C. Schepel , Alzheimers Res. Ther. 2021, 13, 51. 33622392 10.1186/s13195-021-00783-x PMC7901512

[166] J. M. Rho , D. Boison , Nat. Rev. Neurol. 2022, 18, 333. 35361967 10.1038/s41582-022-00651-8 PMC10259193

[167] Y. Su , M. Tang , M. Wang , Aging Dis. 2024, 15, 1289. 37450928 10.14336/AD.2023.0624 PMC11081153

[168] A. M. Planas , Glia 2024, 72, 1016. 38173414 10.1002/glia.24501

[169] H. Wei , L. Zhen , S. Wang , Y. Zhang , K. Wang , P. Jia , Y. Zhang , Z. Wu , Q. Yang , W. Hou , J. Lv , P. Zhang , Neuroscience 2022, 481, 85. 34822949 10.1016/j.neuroscience.2021.11.026

[170] H. Wei , L. Zhen , S. Wang , L. Yang , S. Zhang , Y. Zhang , P. Jia , T. Wang , K. Wang , Y. Zhang , L. Ma , J. Lv , P. Zhang , J. Neuroinflammation 2023, 20, 264. 37968698 10.1186/s12974-023-02942-3 PMC10648711

[171] X. Guan , J. Wu , J. Geng , D. Ji , D. Wei , Y. Ling , Y. Zhang , G. Jiang , T. Pang , Z. Huang , Transl. Stroke Res. 2024, 15, 195. 36577854 10.1007/s12975-022-01121-5

[172] D. Guo , L. Hu , P. Xie , P. Sun , W. Yu , Int. Immunopharmacol. 2024, 134, 112182. 38703568 10.1016/j.intimp.2024.112182

[173] S. Delgado‐Martín , A. Martínez‐Ruiz , FEBS Lett. 2024, 598, 2160. 38676284 10.1002/1873-3468.14894

[174] A. Bezawork‐Geleta , J. Dimou , M. J. Watt , Front. Oncol. 2022, 12, 1085034. 36591531 10.3389/fonc.2022.1085034 PMC9797845

[175] A. Websdale , Y. Kiew , P. Chalmers , X. Chen , G. Cioccoloni , T. A. Hughes , X. Luo , R. Mwarzi , M. Poirot , H. Røberg-Larsen , R. Wu , M. Xu , M. A. Zulyniak , J. L. Thorne , Biochem. Pharmacol. 2022, 196, 114731. 34407453 10.1016/j.bcp.2021.114731

[176] N. Jiang , B. Xie , W. Xiao , M. Fan , S. Xu , Y. Duan , Y. Hamsafar , A. C. Evans , J. Huang , W. Zhou , X. Lin , N. Ye , S. Wanggou , W. Chen , D. Jing , R. C. Fragoso , B. N. Dugger , P. F. Wilson , M. A. Coleman , S. Xia , X. Li , L. Q. Sun , A. M. Monjazeb , A. Wang , W. J. Murphy , H. J. Kung , K. S. Lam , H. W. Chen , J. J. Li , Nat. Commun. 2022, 13, 1511. 35314680 10.1038/s41467-022-29137-3 PMC8938495

[177] P. C. Chang , Y. C. Lin , H. J. Yen , D. Y. Hueng , S. M. Huang , Y. F. Li , Cancer Cell Int. 2023, 23, 62. 37029364 10.1186/s12935-023-02912-y PMC10080956

[178] N. Wang , J. Wang , P. Wang , N. Ji , S. Yue , Anal. Chem. 2023, 95, 11567. 37417930 10.1021/acs.analchem.3c00967 PMC10413324

[179] Z. Hao , J. Wang , Y. Lv , W. Wu , S. Zhang , S. Hao , J. Chu , H. Wan , J. Feng , N. Ji , Metabolism 2024, 153, 155794. 38301843 10.1016/j.metabol.2024.155794

[180] M. De Martino , C. Daviaud , H. E. Minns , A. Lazarian , A. Wacker , A. P. Costa , N. Attarwala , Q. Chen , S. W. Choi , R. Rabadàn , L. B. J. McIntire , R. D. Gartrell , J. M. Kelly , E. C. Laiakis , C. Vanpouille-Box , Cancer Lett. 2023, 570, 216329. 37499741 10.1016/j.canlet.2023.216329

[181] K. Li , Y. Wang , Y. Li , W. Shi , J. Yan , Talanta 2024, 277, 126362. 38843773 10.1016/j.talanta.2024.126362

[182] J. A. Lee , B. Hall , J. Allsop , R. Alqarni , S. P. Allen , Semin. Cell Dev. Biol. 2021, 112, 123. 32773177 10.1016/j.semcdb.2020.07.017

[183] Y. T. Lin , J. Seo , F. Gao , H. M. Feldman , H. L. Wen , J. Penney , H. P. Cam , E. Gjoneska , W. K. Raja , J. Cheng , R. Rueda , O. Kritskiy , F. Abdurrob , Z. Peng , B. Milo , C. J. Yu , S. Elmsaouri , D. Dey , T. Ko , B. A. Yankner , L. H. Tsai , Neuron 2018, 98, 1294. 29953873 10.1016/j.neuron.2018.06.011 PMC6048952

[184] S. Chamberlain , H. Gabriel , W. Strittmatter , J. Didsbury , J. Alzheimers Dis. 2020, 73, 1085. 31884472 10.3233/JAD-190864 PMC7081093

[185] Q. Xu , Y. Zhang , X. Zhang , L. Liu , B. Zhou , R. Mo , Y. Li , H. Li , F. Li , Y. Tao , Y. Liu , C. Xue , Clin. Nutr. 2020, 39, 2092. 31694759 10.1016/j.clnu.2019.10.017

[186] L. Bonfili , M. Cuccioloni , C. Gong , V. Cecarini , M. Spina , Y. Zheng , M. Angeletti , A. M. Eleuteri , Clin. Nutr. 2022, 41, 698. 35158177 10.1016/j.clnu.2022.01.025

[187] M. H. Nam , H. Y. Ko , D. Kim , S. Lee , Y. M. Park , S. J. Hyeon , W. Won , J. I. Chung , S. Y. Kim , H. H. Jo , K. T. Oh , Y. E. Han , G. H. Lee , Y. H. Ju , H. Lee , H. Kim , J. Heo , M. Bhalla , K. J. Kim , J. Kwon , T. D. Stein , M. Kong , H. Lee , S. E. Lee , S. J. Oh , J. H. Chun , M. A. Park , K. D. Park , H. Ryu , M. Yun , et al., Brain 2023, 146, 2957. 37062541 10.1093/brain/awad037 PMC10517195

[188] H. L. Hamilton , N. A. Kinscherf , G. Balmer , M. Bresque , S. M. Salamat , M. R. Vargas , M. Pehar , Geroscience 2024, 46, 1607. 37688656 10.1007/s11357-023-00916-0 PMC10828232

[189] G. S. Green , M. Fujita , H. S. Yang , M. Taga , A. Cain , C. McCabe , N. Comandante-Lou , C. C. White , A. K. Schmidtner , L. Zeng , A. Sigalov , Y. Wang , A. Regev , H. U. Klein , V. Menon , D. A. Bennett , N. Habib , P. L. De Jager , Nature 2024, 633, 634. 39198642 10.1038/s41586-024-07871-6 PMC11877878

[190] J. Alarcon-Gil , A. Sierra-Magro , J. A. Morales-Garcia , M. Sanz-SanCristobal , S. Alonso-Gil , M. Cortes-Canteli , M. Niso-Santano , G. Martínez-Chacón , J. M. Fuentes , A. Santos , A. Perez-Castillo , Cells 2022, 11, 2297. 35892594 10.3390/cells11152297 PMC9331796

[191] O. R. Tamtaji , M. Taghizadeh , E. Aghadavod , A. Mafi , E. Dadgostar , R. Daneshvar Kakhaki , J. Abolhassani , Z. Asem , Clin. Neurol. Neurosurg. 2019, 176, 116. 30554092 10.1016/j.clineuro.2018.12.006

[192] E. Miquel , R. Villarino , L. Martínez‐Palma , A. Cassina , P. Cassina , Glia 2024, 72, 999. 38372421 10.1002/glia.24516

[193] L. Dai , L. Zou , L. Meng , G. Qiang , M. Yan , Z. Zhang , Mol. Neurobiol. 2021, 58, 2183. 33411241 10.1007/s12035-020-02232-6

[194] D. Boison , C. Steinhäuser , Glia 2018, 66, 1235. 29044647 10.1002/glia.23247 PMC5903956

[195] A. Vezzani , T. Ravizza , P. Bedner , E. Aronica , C. Steinhäuser , D. Boison , Nat. Rev. Neurol. 2022, 18, 707. 36280704 10.1038/s41582-022-00727-5 PMC10368155

[196] X. C. Ni , H. F. Wang , Y. Y. Cai , D. Yang , R. N. Alolga , B. Liu , J. Li , F. Q. Huang , Redox Biol. 2022, 54, 102363. 35696763 10.1016/j.redox.2022.102363 PMC9198466

[197] A. Stokowska , M. Aswendt , D. Zucha , S. Lohmann , F. Wieters , J. Morán Suarez , A. L. Atkins , Y. Li , M. Miteva , J. Lewin , D. Wiedermann , M. Diedenhofen , Å. Torinsson Naluai , P. Abaffy , L. Valihrach , M. Kubista , M. Hoehn , M. Pekny , M. Pekna , J. Clin. Invest. 2023;133, e162253. 36995772 10.1172/JCI162253 PMC10178843

[198] M. Arbaizar-Rovirosa , M. Gallizioli , J. J. Lozano , J. Sidorova , J. Pedragosa , S. Figuerola , N. Chaparro-Cabanillas , P. Boya , M. Graupera , M. Claret , X. Urra , A. M. Planas , J. Neuroinflammation 2023, 20, 207. 37691115 10.1186/s12974-023-02888-6 PMC10494365

[199] F. Geng , X. Cheng , X. Wu , J. Y. Yoo , C. Cheng , J. Y. Guo , X. Mo , P. Ru , B. Hurwitz , S. H. Kim , J. Otero , V. Puduvalli , E. Lefai , J. Ma , I. Nakano , C. Horbinski , B. Kaur , A. Chakravarti , D. Guo , Clin. Cancer Res. 2016, 22, 5337. 27281560 10.1158/1078-0432.CCR-15-2973 PMC5093025

[200] X. Cheng , F. Geng , M. Pan , X. Wu , Y. Zhong , C. Wang , Z. Tian , C. Cheng , R. Zhang , V. Puduvalli , C. Horbinski , X. Mo , X. Han , A. Chakravarti , D. Guo , Cell Metab. 2020, 32, 229. 32559414 10.1016/j.cmet.2020.06.002 PMC7415721

